# Advances in Computational Modeling and Machine Learning of Cellulosic Biopolymers: A Comprehensive Review

**DOI:** 10.3390/biomimetics10120802

**Published:** 2025-12-01

**Authors:** Sharmi Mazumder, Mohammad Hossein Golbabaei, Ning Zhang

**Affiliations:** Department of Mechanical Engineering, Baylor University, Waco, TX 76706, USA

**Keywords:** computational modeling, machine learning, cellulosic materials, molecular dynamics simulation, bioinspired materials

## Abstract

The hierarchical structure and multifunctional properties of bio-based cellular materials, particularly cellulose, hemicellulose, and lignin, have attracted increasing attention and interest due to their sustainability and versatility. Recent advances in computational modeling and machine learning strategies have provided transformative insights into the molecular, mechanical, thermal, and electronic behaviors of these biopolymers. This review categorizes the conducted studies based on key material properties and discusses the computational methods utilized, including quantum mechanical approaches, atomistic and coarse-grained molecular dynamics, finite element modeling, and machine learning techniques. For each property, such as structural, mechanical, thermal, and electronic, we have analyzed the progress made in understanding inter- and intra-molecular interactions, deformation mechanisms, phase behavior, and functional performance. For instance, atomistic simulations have shown that cellulose nanocrystals exhibit a highly anisotropic elastic response, with axial elastic moduli ranging from approximately 100 to 200 GPa. Similarly, thermal transport studies have shown that the thermal conductivity along the chain axis (≈5.7 W m^−1^ K^−1^) is nearly an order of magnitude higher than that in the transverse direction (≈0.7 W m^−1^ K^−1^). In recent years, this research area has also experienced rapid advancement in data-driven methodologies, with the number of machine learning applications for biopolymer systems increasing more than fourfold over the past five years. By bridging multiscale modeling and data-driven approaches, this review aims to illustrate how these techniques can be integrated into a unified framework to accelerate the design and discovery of high-performance bioinspired materials. Eventually, we have discussed emerging opportunities in multiscale modeling and data-driven discovery to outline future directions for the design and application of high-performance bioinspired materials. This review aims to bridge the gap between molecular-level understanding and macroscopic functionality, thereby supporting the rational design of next-generation sustainable materials.

## 1. Introduction

Cellulosic biopolymers, readily sourced from the cell walls of trees, coconut shells, plants, and grasses, represent an excellent class of renewable materials. Owing to their sustainable origins and inherent biocompatibility, these natural polymers hold significant value. Their use paves the way for a new generation of materials that not only fulfill the demands of modern applications but also align with the principles of environmental sustainability and biological compatibility [1,2]. The exploration of cellulosic biopolymers for manufacturing advanced, lightweight yet strong materials has enabled applications across the aerospace, automotive, defense, healthcare, and construction sectors [3]. Leveraging the advantages of these natural polymers offers substantial opportunities for the development of innovative materials with enhanced properties [4,5,6,7].

Conducting experimental studies on bio-cellulosic polymers presents challenges due to their hierarchical, inherently complex structure in nature [8,9]. Advanced analytical techniques, such as microscopy, spectroscopy, and rheology, are essential for characterizing the diverse structures and properties of these polymers; however, their implementation at the nanoscale can be technically demanding [10,11,12,13,14,15]. The distinct characteristics of natural materials are often attributed to their nanoscale arrangement, with particular emphasis on the role of interfaces [16,17]. The unique and complementary properties of cellulose, hemicellulose, and lignin, as the dominant cellulosic biopolymers, highlight the importance of focusing on these materials for various industrial and environmental applications [18,19,20].

**Figure 1:**
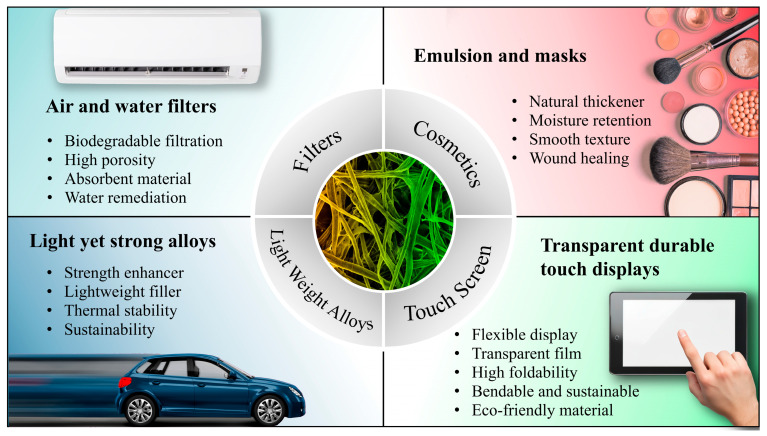
Representative applications of cellulose across various fields.

Cellulose is a fundamental polysaccharide found in the cell walls of plants and serves as a primary structural component of the plant kingdom. Due to its linear crystalline structure, cellulose contributes strength and rigidity to the plant cell wall, making it an excellent candidate for designing biomimetic materials with superior mechanical properties [21,22]. Composed of linear chains of glucose molecules linked by β-1,4-glycosidic bonds [23], cellulose forms robust crystalline microfibrils through intra- and intermolecular hydrogen bonding. This unique arrangement imparts exceptional strength and rigidity to cellulose, contributing to the structural integrity of plant cells. Cellulose has multiple allomorphs; among them, cellulose *Iβ* is of particular interest due to its prevalence in wood, plants, and endocarps. The β–O–4 linkage is among the most abundant and structurally significant inter-unit bonds in lignin, forming the backbone of its complex and heterogeneous structure [24]. In addition to its mechanical properties, cellulose is a renewable and abundant biopolymer with a wide range of applications, as illustrated in Figure 1. It serves as a source of fiber in the textile industry, a key ingredient in paper and cardboard manufacturing, and a promising candidate for biofuel production. The enzymatic breakdown of cellulose is a critical step in the carbon cycle and an area of interest for sustainable biomass utilization.

Hemicellulose, characterized by its branched, heterogeneous structure, contributes to the flexibility and resilience of plant tissues, thereby enhancing the versatility of bio-based materials. It is a heteropolymer composed of various sugars, including xylose, mannose, galactose, and arabinose. In contrast to cellulose and hemicellulose, which are polysaccharides formed from sugar molecules, lignin is an amorphous, aromatic polymer composed of phenylpropanoid units, primarily coniferyl alcohol, sinapyl alcohol, and p-coumaryl alcohol. Moreover, the complex and irregular structure of lignin provides durability and resistance to decay, playing a vital role in maintaining the structural integrity of plant cell walls.

However, the composition of polymers in natural materials, such as wood and coconut endocarp, can vary significantly due to differences in plant species and tissues [25]. Experimental methods alone are often inadequate for capturing the intricate nature of these materials; therefore, computational techniques have become essential tools. The following sections present an overview of these computational methodologies.

**Figure 2:**
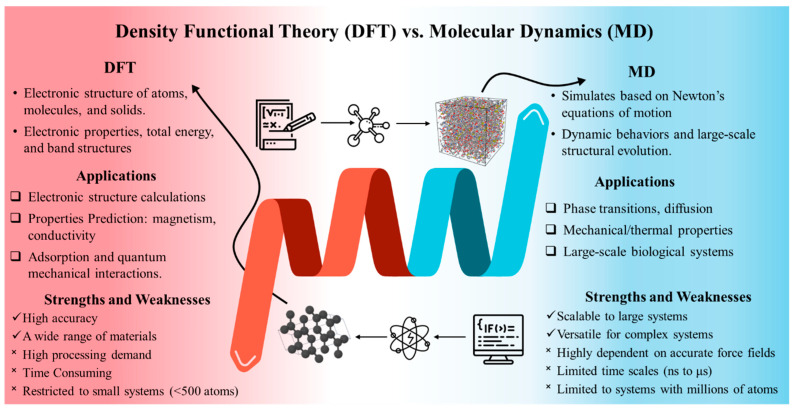
Schematic comparison of MD and DFT, illustrating their respective applications, strengths, and limitations in modeling bio-based polymer systems.

To mimic these biological materials, computational modeling, including advanced methods such as density functional theory (DFT) and molecular dynamics (MD) simulations, is commonly employed to gain insights into their molecular structure, thermal, and mechanical properties [26,27,28,29,30,31]. Figure 2 presents a comparative overview of the two methods, highlighting their respective applications, strengths, and limitations. Given the complex architecture and extended molecular chains of bio-based polymers, DFT exhibits limited capability in capturing their full range of properties. In contrast, MD simulation proves to be a powerful tool for investigating nanoscale features and identifying underlying deformation mechanisms. For instance, it is widely accepted that hydrogen bonding is essential for maintaining the interfacial strength of the cell wall at the nanoscale [32,33]. Through tracking the motion of atoms, MD simulations can capture the dynamic process of hydrogen bond formation and dissociation, reveal polymer folding and unfolding behavior, and enable the investigation of interactions between polymers and solvents or other molecular species [25,34,35].

Additionally, MD simulation plays a pivotal role in unraveling the intricate structure-property relationships of natural polymers. For example, MD studies have revealed that cellulose consists of tightly packed crystalline microfibrils stabilized by extensive intra- and intermolecular hydrogen bonding. This highly ordered structure imparts exceptional tensile strength and rigidity to cellulose, which are essential for the structural stability of plant cells, wood, and the endocarp of nuts and fruits [18,36,37]. Beyond mechanical reinforcement, MD simulations provide a comprehensive analysis of the physical and chemical characteristics of cellulose, including unit cell parameters, density, hydrogen bonding networks, van der Waals interactions, thermal behavior, and interactions with solvents such as water and benzene [34,38]. While further investigation is required to fully understand the complex architecture of cellulose, lignin, and the composite nature of plant cell walls [2,39], MD provides valuable insights into how specific linkages affect the conformational preferences of lignin and its interactions with water molecules [40]. The versatility of MD simulations is further underscored by their application to various cellulose polymorphs, including Iα, Iβ, II, para-crystalline cellulose, and amorphous cellulose [41,42], providing a robust computational toolkit for advancing our understanding of natural polymer systems.

While MD is effective at the molecular level, continuum-level approaches, such as finite element modeling (FEM), provide complementary insights by analyzing material behavior at larger scales. FEM is a powerful numerical method that divides a complex system into smaller, manageable subdomains called finite elements, enabling the solution of governing equations across these discrete units. This approach has been widely employed to predict material properties and investigate the behavior of fibrous and cellulosic materials under various conditions [43,44]. FEM has proven useful in simulating the mechanical response of cellulosic materials [45], performing multiscale analyses of cellulose-reinforced polymer composites [46], and modeling moisture absorption behavior in bulk cellulosic structures [47]. Unlike DFT or MD, FEM is capable of modeling macroscopic behaviors and has demonstrated significant potential over several decades in materials research.

**Figure 3:**
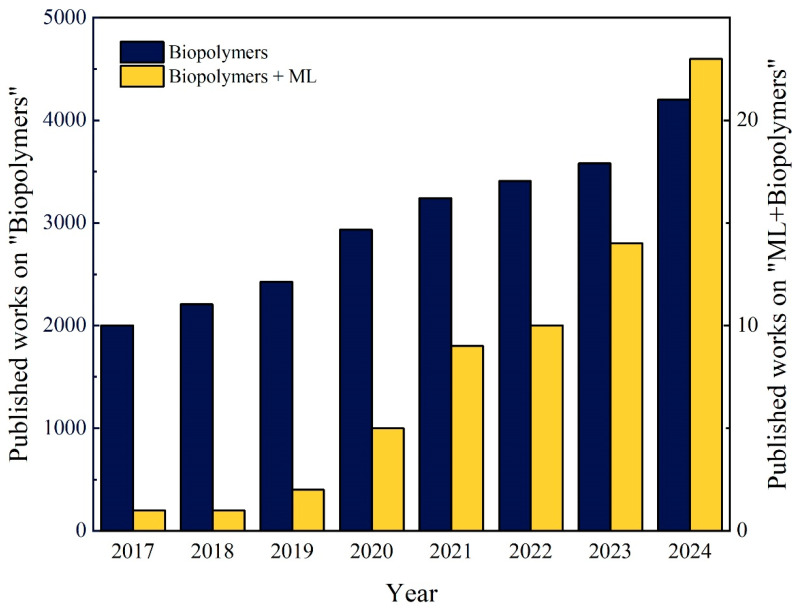
Trends in the number of published works related to “Biopolymers” and the combined topic “Biopolymers + Machine Learning” from January 2017 to December 2024. Publication data were retrieved from the Scopus database using the Title, Abstract, and Keywords search fields and analyzed by the authors.

Complementary to both molecular and continuum simulations, machine learning (ML), a subfield of artificial intelligence (AI), offers a promising paradigm for accelerating discovery and decision-making in materials science. ML enables systems to learn from data without explicit programming or prior domain-specific knowledge, making it particularly well-suited for solving complex, non-linear problems, as well as for optimization and prediction tasks [48]. ML has emerged as a powerful tool in materials engineering, contributing to performance prediction [49], materials design [50], and the discovery of novel compositions [51]. It supports both supervised and unsupervised learning strategies, including regression, classification, and clustering techniques [52]. In the context of biopolymers, several studies have employed ML models such as Artificial Neural Network (ANN), Gaussian Process (GP), and Support Vector Machine (SVM) to accelerate research and reduce experimental workload. The complex microstructures and unique behaviors of biopolymers necessitate the use of advanced analytical and computational approaches. The growing interest in this field is reflected in the increasing number of publications on the integration of biopolymers and ML from January 2017 to December 2024, based on data extracted from the Scopus database and analyzed by the authors (Figure 3). The search procedure used to construct Figure 3 followed a reproducible querying strategy, which is described in the caption, and publication counts were extracted directly from the Scopus “Analyze Results” function to avoid duplicate counting. As illustrated, the application of ML in biopolymer research is not only growing but also emerging as a transformative and innovative direction. Given the rapid advancements in AI and data-driven science, there remain substantial opportunities for further exploration and innovations in this field.

This review summarizes recent advancements in computational techniques, including DFT, MD, FEM, and ML, as applied to biopolymers such as cellulose, hemicellulose, lignin, and their composites. A comprehensive understanding of the multiscale mechanisms governing these cellulosic materials serves as a foundation for the tailored design and optimization of their properties for diverse applications. Moreover, although several existing review articles discuss either computational modeling or machine learning in the context of cellulose and lignocellulosic materials, these works typically consider the two approaches in isolation and do not address how insights may be transferred between them. As noted earlier, the unique contribution of this review work is the integration of a multiscale modeling perspective, spanning quantum-level DFT and atomistic MD to continuum-scale FEM and data-driven ML frameworks, to illustrate how knowledge generated at one scale can inform decision-making at other scales. The goal of this review is to conceptually bridge these approaches by highlighting the interdisciplinary relationship between physics-based simulation and ML-based prediction and optimization, forming a unified framework for the accelerated design, processing, and evaluation of next-generation cellulosic materials. This framework is designed to facilitate the systematic development of hierarchical structure–property relationships.

## 2. Computational Techniques

Hierarchical materials, such as biological tissues or composite materials, are characterized by intricate arrangements of components across multiple length scales, from the nanoscale to the macroscale. Since no single modeling approach can capture all relevant phenomena across scales, multiscale modeling becomes essential. As simulation techniques complement experimental efforts, they offer a cost-effective and time-efficient means to explore molecular interactions, structural evolution, and thermodynamic behavior. In particular, cellulose, with its complex and hierarchical molecular architecture, derives significant benefit from computational studies.

**Figure 4:**
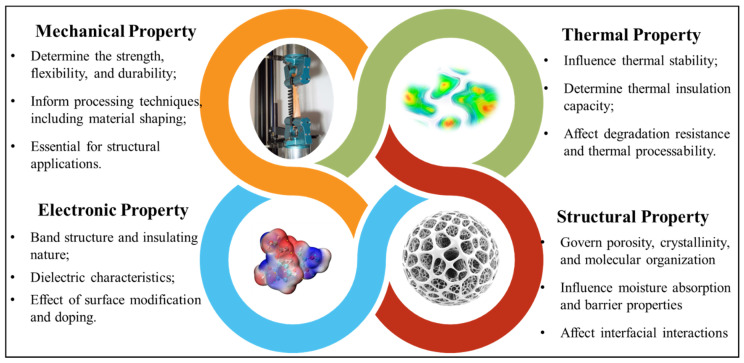
Key properties relevant to cellulosic materials and their significance in determining performance and functionality.

At the nanoscale, phenomena such as molecular interactions, quantum effects, and atomic arrangements are of critical importance. Classical DFT calculations and MD simulations are well-suited for investigating these fundamental aspects [18,53,54]. At larger scales, coarse-grained (CG) modeling, continuum-based finite element analysis (FEA), and other macroscopic approaches become necessary to describe the mechanical behavior and interactions of larger structural features. To provide a comprehensive overview, these computational techniques are discussed in detail, along with their specific applications in property prediction and analysis. Key properties, including mechanical, thermal, structural, optical, electronic, and catalytic characteristics, have been investigated by these methods. Figure 4 provides a comprehensive illustration of the primary properties and highlights the corresponding computational approaches used in their investigation.

### 2.1. Density Functional Theory (DFT)

DFT is a powerful approach for studying the structural, electronic, and vibrational properties of cellulosic materials. By modeling electron density rather than wavefunctions, DFT provides quantum-level insights into band structures, charge distributions, and bonding characteristics in complex biopolymers. The Kohn-Sham equations approximate electron interactions using an effective potential derived from the electron density. Hybrid functionals, such as B3LYP, X3LYP, M06, and TPSSh, are widely used, with B3LYP being the most commonly employed. While DFT is computationally demanding, it provides high accuracy, though the results can vary depending on the chosen functionality and the complexity of the system.

### 2.2. Molecular Dynamics (MD)

Building on DFT insights at the electronic level, MD simulations extend the analysis to the molecular and nanoscales, providing essential tools for investigating the structural, mechanical, and dynamical behavior of biopolymers. MD reveals key features such as conformational flexibility, hydrogen bonding networks, and degradation mechanisms under various environmental conditions, which are critical for applications in biofuels, biomaterials, and sustainable composites. By employing biopolymer-specific force fields, MD accurately captures intra- and intermolecular interactions and can be integrated with experimental observations to guide the rational design of advanced materials.

Early MD researchers developed force fields as efficient tools for defining the potential energy landscape. Classical force fields, such as AMBER (GAFF) [55], PCFF [56], OPLS-All-Atom (OPLS-AA) [57], GROMACS [58], and CHARMM [59], are widely used for predicting mechanical and thermodynamic properties, binding affinities, and structural responses of systems under varying conditions. To explore polymer interfacial strength, particularly between crystalline and amorphous regions, advanced simulation techniques, including free-energy perturbation [60], umbrella sampling [61], replica exchange [62], metadynamics [63], steered molecular dynamics (SMD) [64], and accelerated molecular dynamics [65], have significantly enhanced our understanding of dynamic processes at the atomic scale.

In general, MD simulations can be categorized into all-atom (AA) and coarse-grained (CG) models. The selection depends on system complexity, resolution requirements, and computational capacity. AA simulations are preferred when detailed atomic interactions are required, particularly for small systems over short timeframes. In contrast, CG simulations reduce computational cost and are better suited for capturing large systems over longer timescales [66]. CG approaches have significantly advanced the study of carbohydrates by revealing their structural flexibility, interaction patterns, and self-assembly mechanisms [67,68]. Although converting atomistic models to CG representations presents challenges, established methods such as inverse Monte Carlo (IMC), iterative Boltzmann inversion (IBI), and force matching (FM) facilitate this process.

### 2.3. Finite Element Method (FEM)

Transitioning from the molecular to the continuum scale, the FEM serves as a powerful computational tool for modeling the macroscopic mechanical behavior of biopolymers. FEM is particularly useful in the study of natural fiber-reinforced composites, biodegradable packaging materials, and soft bio-based structures, allowing the analysis of stress–strain responses, fracture mechanics, and viscoelastic properties of cellulose fibers. By integrating FEM with AA and CG MD simulations, researchers can effectively bridge nanoscale interactions with macroscopic material behavior, offering a comprehensive multiscale understanding of cellulose-based systems. This hybrid approach has been widely applied to study key characteristics related to biodegradability, offering critical insights into the structural integrity and environmental performance of nanocellulose composites, biodegradable polymers, and bio-inspired materials.

### 2.4. Machine Learning (ML)

Complementary to these physics-based techniques, ML, a core subfield of AI, is reshaping materials research by enabling faster discovery and deeper mechanistic understanding. ML algorithms identify patterns and develop predictive models based on existing datasets, learning iteratively through optimization techniques guided by loss functions. Within ML, deep learning (DL), which is primarily based on ANNs, is particularly powerful, while traditional algorithms such as K-nearest neighbors (KNN), linear models, decision trees, SVM, and GP remain widely used and effective.

The integration of ML with simulation techniques has led to significant advances in both industry and academia. ML now plays a pivotal role in materials design, property prediction, performance assessment, and process optimization [69]. A particularly impactful application is the development of ML-driven interatomic potentials for MD simulations, which has substantially enhanced computational efficiency across diverse material systems [70,71,72]. Furthermore, MD-generated datasets can be utilized by ML models, such as generative adversarial networks (GANs), to create new data, forming a feedback loop that continuously improves model accuracy and accelerates materials innovation [73].

## 3. Computational Investigation of Cellulosic Material Properties

To date, computational methods have been extensively applied to study and predict the mechanical, thermal, optical, structural, electronic, and catalytic properties of cellulose and related biopolymers. These approaches provide valuable, high-throughput insights across multiple length scales, from atomic-level interactions to macroscopic behavior. In this section, we present selected case studies that illustrate the application of these computational techniques in advancing the understanding and design of cellulose-based systems.

### 3.1. Structural Properties

The structural characteristics of cellulose, including chain orientation, crystallinity, hydrogen bonding, and hierarchical organization, are fundamental to its mechanical performance, chemical reactivity, and functional versatility in various applications, ranging from sustainable composites to biomedical scaffolds. Given the complex multiscale architecture of cellulose, an integrated computational approach is essential to fully characterize and optimize its structural behavior.

DFT provides highly accurate insights into the electronic structure and chemical bonding of cellulose at the atomic scale. MD reveals how hydrogen bond networks evolve, how chains realign under mechanical stress, and how crystalline and amorphous domains interact, all of which contribute to understanding the stiffness, flexibility, and deformation mechanisms of materials. CGMD extends these capabilities to larger structures such as fibrils, bundles, and polymeric networks by simplifying molecular details while preserving essential physical interactions. This enables efficient simulation of mesoscale behavior, including fiber assembly, bending response, and network mechanics under various environmental conditions. At the macroscopic level, FEM captures the influence of microstructural features, such as porosity, fiber orientation, and interface quality, on stress distribution, stiffness, and failure mechanisms, many of which are challenging to isolate experimentally. Finally, ML complements these physics-based methods by identifying hidden patterns and correlations within large-scale experimental and simulation datasets, enabling rapid predictions of structural behavior and design optimization. Among these approaches, MD simulations have made especially significant contributions to the structural characterization of cellulosic materials. Therefore, the following discussions will focus primarily on MD-based studies in this domain.

#### 3.1.1. Atomistic Simulations (AAMD)

AAMD simulations using the GLYCAM-06 force field were conducted to explore the structural and thermal behavior of cellulose Iβ [74]. It was found that the C_1_–O_4_ bond is stronger than the C_4_–O_4_ bond, which acts as the primary failure site in CNCs, regardless of twist direction. Although hydrogen bonds are significantly weaker than covalent bonds and do not directly govern failure, they play a crucial role in maintaining structural stability. Two distinct transition temperatures, ~350 K and ~450 K, were identified, with the latter consistent with experimental data. Temperature-induced structural changes included chain twisting, hydroxymethyl group reorientation, hydrogen bond disruption, and inter-chain sliding. During the regeneration process, cellulose chains stack along hydrophobic faces and are stabilized by hydrogen bonding on hydrophilic sides, initiating cellulose II crystallization and self-assembly [75].

**Figure 5:**
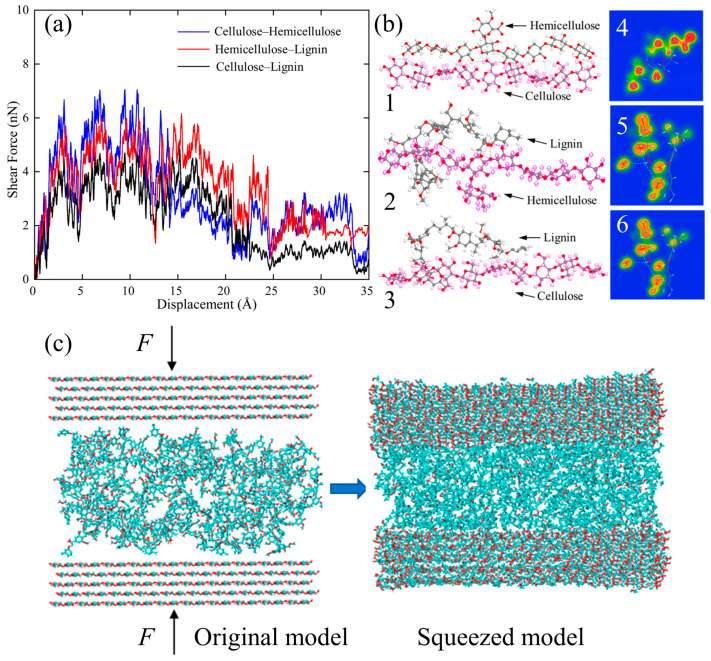
(**a**) Force–displacement curves measured during the SMD shear simulations. (**b**) Adsorption sites and H-bonds formed between (1) cellulose–hemicellulose, (2) hemicellulose–lignin, and (3) cellulose–lignin. The electron CDD of (4) cellulose–hemicellulose, (5) hemicellulose–lignin, and (6) cellulose–lignin couples. (**c**) Molecular model of the sandwiched structure of cellulose-hemicellulose-lignin in coconut shell, highlighting the interfaces between them [32]. Reproduced under the terms of the Creative Commons Attribution (CC BY 4.0) license.

The strength of cellulosic material, such as wood and coconut shell, is primarily rooted in their nano- and submicron-scale organization, where polymer chains are assembled into fibrillar structures embedded within a surrounding matrix [76]. Early research aimed at understanding plastic deformation mechanisms relied largely on microscale experimental techniques. However, atomistic simulations of coconut endocarp indicated that the cellulose–hemicellulose interface exhibits greater interfacial strength than the cellulose–lignin interface, primarily due to the lower oxygen density of lignin, which limits the formation of hydrogen bonds [32]. To thoroughly investigate these interactions, steered molecular dynamics (SMD) simulations were carried out on couples of polymers to produce serrated force—displacement response related to cycles of hydrogen-bond formation and rupture. As illustrated in Figure 5a, cellulose—hemicellulose has the highest peak shear force while cellulose—lignin has the lowest. These findings were also confirmed by DFT adsorption energies, and the respective hydrogen-bond architectures and electron density distributions are shown in Figure 5b, which clearly shows that the cellulose—hemicellulose has the greatest number of interfacial hydrogen bonds while cellulose—lignin has the least. To further understand these interfacial interactions, uniaxial shear and tensile simulations were performed on layered polymeric models (Figure 5c). The results imply a hierarchical organization of the coconut endocarp where cellulose fibrils are encapsulated by hemicellulose and then embedded in a lignin rich matrix, in agreement with its established high lignin content.

**Figure 6:**
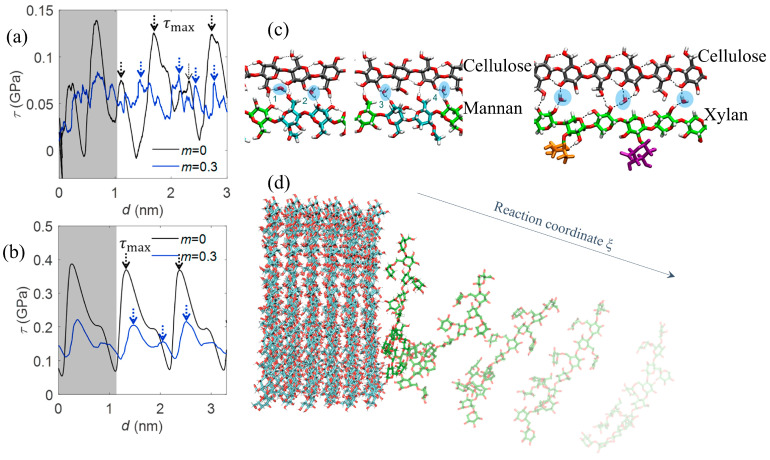
Interfacial mechanics and molecular interactions in cellulosic nanocomposites. Shear stress–displacement curves for softwood cellulosic nanocomposite under (**a**) unrestrained and (**b**) restrained conditions. Black and blue curves represent moisture content m ∼0 and 0.3, respectively; the gray-shaded region indicates the first loading cycle [77]. (**c**) Cross-sectional snapshot of a solvated MD simulation showing mannan–cellulose and cellulose–xylan complexes, reproduced under the terms of the CC-BY-NC-ND 4.0 license from [25]. (**d**) Pulling simulations of hemicellulose molecules along the reaction coordinate from a bound configuration to investigate unbinding mechanisms relative to the cellulose nanocrystal surface [78], reproduced under the terms of the CC BY 4.0 license.

MD simulations have also been used to study interfacial mechanics in cellulosic composites. They revealed that the stick-slip behavior at the fiber-matrix interface is governed by hydrogen bonding dynamics (Figure 6a,b) [29,77]. Another critical application of MD is the study of thermal depolymerization, which may begin with random chain scission events triggered by increased atomic vibrations and localized bond weakening [25]. As shown in Figure 6c, temperature-induced fluctuations in local structure facilitate the formation of volatile fragments and char precursors. Moreover, MD has been employed to simulate the evolution of gaseous products during cellulose thermal degradation [78]. For instance, Figure 6d illustrates the release of H_2_O, CO, CO_2_, CH_4_, and other small molecules at various high temperatures, offering insight into how reaction rates and product distributions vary with thermal intensity.

Beyond structural changes, MD enables exploration of the detailed reaction pathways involved in cellulose pyrolysis. By using ReaxFF-based reactive MD, Jin et al. [37] mapped the sequential bond-breaking and bond-forming events, which lead to the formation of key degradation products, such as levoglucosan, water, and various gases. Their study highlighted how thermal energy initiates glycosidic bond cleavage and subsequent rearrangements, providing a dynamic perspective of intermediate structures and reaction mechanisms. Furthermore, MD simulations have quantified the effects of moisture content and matrix deformability on interfacial shear stress, demonstrating a strong correlation between hydrogen bond density and stress transmission. These insights, inaccessible by experimental techniques alone, demonstrate the unique power of atomistic MD in capturing the dynamic, moisture-sensitive interfacial phenomena, which are critical to the mechanical performance of natural fiber composites.

In an MD simulation of native cellulose [79], two distinct crystalline forms were identified: the two-chain monoclinic phase (cellulose Iβ) and the single-chain triclinic phase (cellulose Iα). The results indicated that cellulose Iβ is thermodynamically more stable than Iα by −8.7 kJ mol^−1^ per cellobiose unit, a finding that aligns well with experimental observations. Structural analysis revealed that in cellulose Iβ, glucose residues in alternating molecular layers are oriented parallel to the (200) plane, promoting favorable intermolecular hydrogen bonding. In contrast, cellulose Iα lacks this alignment, resulting in weaker bonding interactions. Further simulations [80] demonstrated that molecular mobility, fractional free volume, and diffusivity of cellulose are strongly influenced by temperature, the presence of water, and the degree of ring opening. The conversion of cellulose to dialcohol cellulose led to enhanced chain mobility under thermal conditions relevant to thermoplastic processing (above *T_g_*), while exhibiting minimal influence on mechanical behavior at room temperature.

Additionally, the binding affinity of Li^+^ ions to cellulose, verified through NMR diffusometry [81], was found to be consistent with MD simulation results [82], supporting the presence of direct interactions between cations and cellulose hydroxyl groups. Although the hierarchical sheet strength of cellulose remained relatively stable across different temperatures, the toughness of the material, estimated from strain energy at fracture, exhibited significant temperature sensitivity, indicating increased brittleness at elevated temperatures [83].

#### 3.1.2. Coarse-Grained Molecular Dynamics (CGMD)

In order to simulate larger systems over extended timescales, CGMD can outperform classical MD in terms of computational efficiency and cost, albeit at the expense of reduced structural detail. CG models represent atomistic structures using a reduced number of descriptors, where groups of atoms in a molecule or system are mapped to single particles, referred to as beads or interaction sites. However, one major challenge in CGMD is the mapping strategy itself. Since there is no universally accepted method, this process is often guided by chemical intuition and prior experience [84].

**Figure 7:**
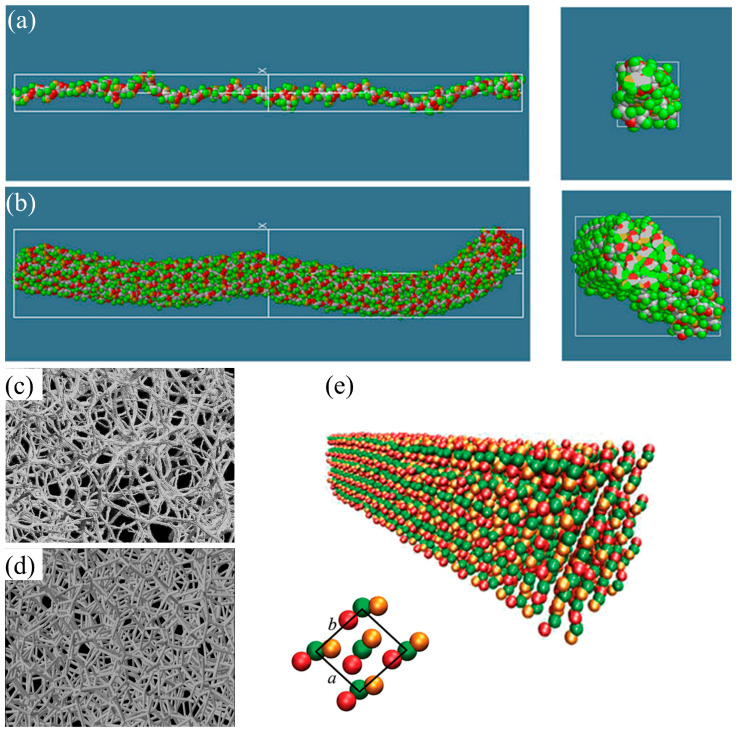
Atomic and molecular configuration of CNF and CNF-MFs and related CG models. Side view and cross-sectional representations of (**a**) single CNF and (**b**) a 3-layered CNF-MF after 40 ps of structural relaxation at a constant temperature of 10 K, reprinted under the terms of the CC-BY 4.0 license from [85]. Comparison of aerogel microstructure generated using (**c**) a DEM-based gelation model, and (**d**) a reconstructed structure via Voronoi approach, reprinted under the terms of the CC-BY 4.0 license from [86]. (**e**) Snapshot of a stable CG cellulose crystal structure and corresponding representation of the cellulose Iβ crystal unit cell. The crystallographic axes a and b are also indicated in the figure, the third crystal axis c is directed parallel to the fibril long axis, which is pointing out from the figure to the right. Reprinted from [68], Copyright 2011 American Chemical Society.

One study on cellulose nanofiber microfibrils (CNF-MFs) employed a united-atom modeling methodology to investigate diffusion behavior. The results revealed that the diffusion coefficient decreased as the number of fibril layers increased, suggesting higher structural stability in multi-layer CNF-MFs. Additionally, structural relaxation analysis demonstrated CNF-MFs spontaneously twisted and stabilized at an average twist angle of 16° (Figure 7a,b) [85]. Jarms et al. [86] developed a CGMD-based simulation framework for modeling gelation and structure formation in cellulose aerogels (Figure 7c,d). Using a discrete element method (DEM) combined with Langevin dynamics, they analyzed the evolution of fibrillar networks and solvent-driven morphological changes. The simulation outcomes were consistent with experimentally observed porosity trends and validated the capability of CGMD in investigating processing–structure relationships in biopolymer aerogels.

Tang et al. [87] employed CGMD to study the molecular-level strengthening mechanisms in high-performance W-paper, which is derived from chemically treated natural wood. By modeling the wood cell wall as a composite of cellulose, hemicellulose, and lignin, they investigated how the removal of amorphous lignin and hemicellulose affects mechanical behavior under tensile stress. Simulation results revealed that the tensile strength is primarily influenced by the stretching and sliding of crystalline cellulose bundles, while hemicellulose and lignin played a more passive role. As the amorphous content decreased, the system exhibited stronger inter-fibrillar bonding and improved crystallinity, resulting in enhanced mechanical performance. These findings highlighted the critical role of dual-phase nanostructure regulation in reinforcing bio-based materials.

The Martini force field is widely used to assign a valid and practical potential for CG simulations, especially for cellulose and other polysaccharides [88]. Wohlert et al. [68] developed a Martini-based CG model for native cellulose, which successfully reproduced solvation free energies and stabilized the cellulose Iβ crystal structure. Their simulations captured key structural features, such as staggered chain packing and chain orientation, and enabled the modeling of protein–cellulose interactions at the microsecond scale. For example, simulations of the carbohydrate-binding domain of Cel7A on cellulose surfaces revealed stepwise diffusion in agreement with experimental data, underscoring the practical application of CGMD for studying cellulose interfacial dynamics (Figure 7e).

### 3.2. Mechanical Properties

Many cellulose crystals and nanocrystals are utilized in bundled forms; therefore, assessing their mechanical properties as a function of twist angle and interfacial energy is critical for understanding and optimizing their performance. Depending on the system size, level of detail required, and available computational resources, two main approaches are employed for such analyses: AAMD and CGMD.

#### 3.2.1. Classic AAMD Simulations

AAMD provides atomistic-level resolution and has been extensively used to investigate the mechanical properties of cellulose in both crystalline and amorphous forms. Compared to the crystalline phase, amorphous cellulose exhibits a significant reduction in elastic modulus, hydrogen bonding density, and bond energy, all of which substantially influence mechanical behavior. Notably, studies have identified an intermediate structural phase, termed “paracrystalline,” that exists between crystalline and amorphous cellulose. This crystalline-to-amorphous transition typically occurs between 177 °C and 277 °C, accompanied by pronounced changes in hydrogen bonding networks and lattice parameters [41].

In addition, AAMD simulations have elucidated the molecular deformation mechanisms of cellulose, hemicellulose, and lignin, revealing two distinct regimes: initial yielding of the matrix, followed by the matrix sliding along cellulose surfaces. These findings provide detailed atomistic insights into the stress-strain responses and interfacial interactions within cellulosic composites [37]. Furthermore, studies have demonstrated that the mechanical properties of cellulose bundles, such as elastic modulus and tensile strength, decrease with increasing twist angle [29]. However, enhancements in interfacial energy can mitigate these effects, leading to improvements in elastic modulus, strength, and toughness. It has also been shown that larger cellulose bundles undergo greater mechanical degradation, underscoring the importance of bundle size in material design [89].

Investigations using AAMD revealed that anions play a critical role in disrupting the hydrogen-bonding network within cellulose structures, increasing strand flexibility while causing structural destabilization. Specifically, anions bind strongly to hydroxyl groups, weakening inter-strand hydrogen bonds, whereas cations intercalate between strands. Rabideau et al. [90] highlighted the significant impact of cation-anion interactions on dissolution efficiency, emphasizing the need for optimizing ionic liquid-based solvents for cellulose processing.

**Figure 8:**
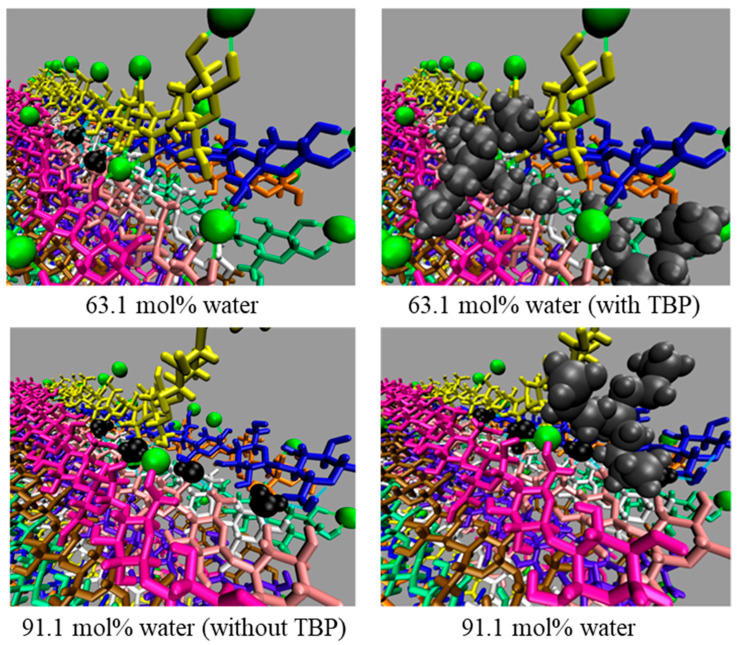
Molecular snapshots of cellulose dissolution in TBPCl–water systems at 63.1 mol% (45 ns) and 91.1 mol% (191 ns) water concentration, with and without TBP. Green dashed lines indicate Cl^−^–cellulose hydrogen bonds; light blue dashed lines denote water––cellulose hydrogen bonds [91], reproduced under the terms of the CC BY 4.0 license.

Further insights were gained through the study of cellulose dissolution in the tetrabutylphosphonium chloride (TBPCl)–water system (Figure 8). AAMD simulations demonstrated that chloride anions initiate dissolution by breaking intra- and inter-strand hydrogen bonds, while TBP cations stabilize the separated strands. Hydrogen-bond lifetime analyses revealed that, for effective dissolution, chloride-cellulose interactions must exceed the strength of intrinsic intra-strand hydrogen bonds. These findings advance the molecular-level understanding of cellulose solvation mechanisms and underscore the need to optimize ionic liquid compositions to enhance cellulose dissolution for biofuel and advanced material production [91].

Following dissolution, cellulose can regenerate into new structural arrangements depending on environmental conditions. Dissolved chains first aggregate into monomolecular sheets via hydrophobic interactions and subsequently form larger crystallites or fiber-like structures, predominantly adopting the thermodynamically favorable cellulose II allomorph. Utilizing CG simulations, researchers can further design efficient cellulose processing and recycling strategies by elucidating the dissolution–reassembly cycle of cellulose [92].

Reactive MD simulations of cellulose nanocrystals have provided valuable atomic-scale insights into their mechanical behavior and failure mechanisms. These studies demonstrated that the C_4_–O_4_ glycosidic bonds are the primary failure sites, while hydrogen bonds play a supporting role in maintaining structural integrity [54]. At low strain rates, these effects stabilize, and the twist orientation of cellulose nanocrystals does not alter the failure mechanism. However, twisting has a significant influence on the mechanical properties of cellulose microfibrils. Microfibrils naturally develop a stable, right-handed twist, with the twisting angle inversely proportional to their cross-sectional area, i.e., smaller microfibrils exhibit greater twisting [93].

Orientation-dependent behavior is another critical factor in cellulose mechanics. In Iβ cellulose, the mechanical properties are highly anisotropic, with the axial modulus substantially exceeding the transverse moduli. Tensile strength and failure strain are highest along the chain axis, whereas deformation in the transverse direction is primarily governed by hydrogen bond reorganization and slip mechanisms [94].

One key application of cellulose fibers and nanocrystals is as reinforcing agents in composite materials [95]. MD simulations have been extensively used to investigate their reinforcing effects. For example, cellulose nanocrystals significantly enhance the tensile strength and ductility of ceramics, particularly cement, by forming strong interfacial interactions with calcium-silicate-hydrate (C-S-H), the primary binding phase in cementitious materials. The calculated interfacial energy between cellulose and C-S-H is approximately −1.09 J/m^2^, which is substantially higher than that of carbon nanotube (CNT)–C-S-H interactions, indicating that nanocellulose is a superior reinforcement material. This enhancement is largely attributed to the hydrogen-bonding network between cellulose and C-S-H, which improves fracture resistance and enables crack-bridging effects [96]. Further improvements in cementitious systems have been achieved by combining CNCs with polyethylene (PE). CNCs with carboxyl functionalization (CNC-C) promote better cement hydration and increase the compactness of the C-S-H gel, while PE coatings increase surface roughness, enhancing mechanical interlocking with the matrix and improving mechanical properties [97].

Beyond ceramics, cellulose-based materials have been broadly incorporated into polymer composites. MD simulations of cellulose blended with polylactic acid (PLA) revealed that cellulose substantially increases the Young’s modulus compared to pure PLA. Cellulose acts as a load-bearing phase, reducing PLA molecular chain stretching and enhancing composite strength. Tensile testing at the atomic scale showed that yielding initiates through infiltration of free volume, followed by void formation and expansion under applied strain [42]. These atomic-scale simulations offer critical insights that are difficult to obtain experimentally, thereby advancing the rational design of high-performance cellulose-reinforced composites across a range of material systems.

#### 3.2.2. CGMD Simulations

On the other hand, CGMD simulations have been instrumental in advancing the understanding of cellulose mechanical behavior across various structural levels, including single fibrils, fibril bundles, and cellulose networks. For instance, studies employing the Martini CG model [98] calculated the axial elastic modulus of a hundred-mer (100 monomer units) cellulose fiber, reporting a value of approximately 10 GPa for a 36-chain fiber. However, direct comparison between simulation results and experimental data remains challenging due to variations in fiber type, diameter, and measurement techniques. Nevertheless, these studies highlight the significant impact of fiber origin and structural characteristics on mechanical performance. Additionally, the potential underestimation of electrostatic interactions in computational models may partly explain the lower elastic moduli observed in some simulations.

Young’s modulus values for single fibrils of cellulose Iβ have been assessed both longitudinally (*Eₙ*) and transversely (*Eₜ*) using various CG models [99,100]. Predictions using the REACH CG model yielded *Eₙ* values of 110–162 GPa and *Eₜ* values of 7–41 GPa, aligning well with experimental measurements (93–220 GPa). Similarly, a continuum-based approach by Shishehbor et al. [99] reported *Eₙ* ≈ 147 GPa and *Eₜ* between 6.5–19 GPa, further supporting the reliability of CG simulations. Notably, the REACH model captured the temperature dependence of cellulose mechanics, demonstrating a linear decrease in *Eₙ* with rising temperature.

**Figure 9:**
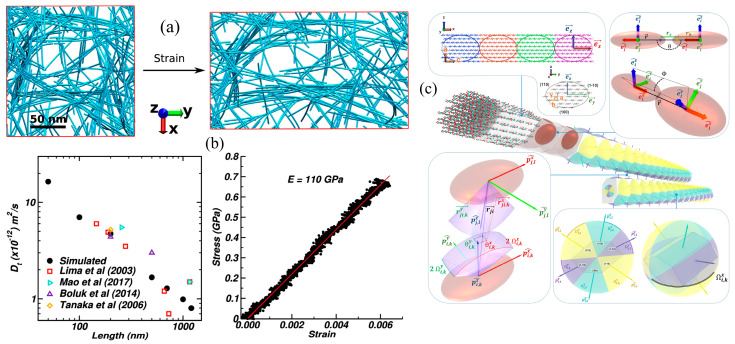
(**a**) Comparison of calculated and experimental diffusion coefficients for cellulose systems [103], reproduced under the terms of the CC BY 4.0 license. (**b**) Simulation snapshots showing the system prior to deformation and after uniaxial deformation along the y-axis at a rate of 1 $\times \;10^{-3}$ nm/ps [103], reproduced under the terms of the CC BY 4.0 license. (**c**) Self-assembled structures of cellulose as captured in CGMD simulation snapshots, illustrating hierarchical organization and chirality-driven ordering. Reprinted from [102], Copyright 2020 American Chemical Society.

Chibrikov et al. [101] developed a CGMD model for investigating the mechanical behavior of bacterial cellulose–hemicellulose composites. They employed a bead–spring representation for fibers and simulated uniaxial tensile deformation to investigate how fiber-level parameters, such as modulus, diameter, length, and interfiber adhesion, can affect network stiffness and strength. The strong agreement between their simulated and experimental stress–strain responses demonstrated how different hemicelluloses (xylan, arabinoxylan, xyloglucan, and glucomannan) influence the mechanical properties of the composite by modifying interfiber interactions and fiber structure. In another study, Rolland et al. [102] developed a novel patchy particle CG model for simulating the self-assembly and mechanical properties of CNCs (Figure 9c). They extended the traditional Kern–Frenkel model with anisotropic patch geometries and captured the interaction between CNC facets. Their model was parameterized against all-atom MD simulations and validated by reproducing the elastic properties of CNCs. They computed a longitudinal elastic modulus of ~340 GPa for single CNCs using steered MD, which represents the behavior of a fully crystalline, defect-free nanocrystal under ideal alignment. Therefore, this obtained value represents the theoretical upper bound. In contrast, to obtain experimental elastic moduli, bundles, films, or fibers with microfibril misalignment, amorphous regions, hydration effects, and structural defects are generally used, and all these defects reduce effective stiffness. Hence, the resulting difference between the computational and experimentally measured values stems from the difference between idealized atomistic models and the nature of real cellulosic materials. Additionally, their model successfully simulated the formation of cholesteric ribbons and tactoids, and reproduced chirality-driven ordering observed in experiments.

While the majority of studies focus on individual or isolated fibers, Mehandzhiyski et al. [103] developed a supra-CG (sCG) model of CNCs to enable large-scale simulation of mechanical and self-assembly behavior. They employed force-matching and umbrella sampling based on AAMD data, parameterized bonded and non-bonded interactions for CNCs, and validated the model with both experiments and atomistic simulations. To evaluate mechanical properties, they conducted uniaxial tensile simulations and calculated an elastic modulus of ~110 GPa for an isolated CNC fibril, which was in good agreement with experimental values. Beyond single fibers, they also modeled the deformation behavior of CNC hydrogels formed via simulated solvent evaporation and found the elastic moduli of 7.2–12.7 MPa, consistent with experimental values for cellulose-based hydrogels (Figure 9a,b).

CGMD simulations have been extended to more complex structures. Qin et al. [104] investigated staggered CNC architectures, revealing that CNC length (10–100 nm) critically influences mechanical properties, such as elastic modulus, toughness, and strength. In bulk disordered nanocellulose networks, CG simulations revealed that increasing the density and cohesive interactions between CNCs enhances both the shear modulus and yield stress. These mechanical improvements are strongly governed by the cohesive energy density of the system. Interestingly, dynamic heterogeneity effects were observed, with the networks exhibiting glass-forming polymer-like behavior, in which increasing density amplified local stiffness variations [105]. For CNC bundles, CG studies demonstrated that improving interfacial properties initially enhances elasticity but reduces toughness, with an optimal performance occurring at a specific level of interfacial strength, highlighting the importance of twisted CNC/CNC interfaces [106].

CG modeling has also been employed to investigate ordered structures and mesoscale interactions in cellulose [107]. A directional hydrogen-bonding CG model successfully captured critical features such as hydrogen-bonding networks, interchain distances, and interchain orientations. These simulations demonstrated how solution conditions and chemical modifications (e.g., methylation) profoundly impact cellulose morphology. Finally, CGMD simulations have illuminated the mechanical transition from elastic to plastic deformation in cellulose networks, showing a strong dependence on the force constant of inter-fiber interactions. Modifying the inter-fiber contact strength or number led to significant changes in network stiffness and elastic behavior, whereas the influence of fiber modulus and diameter on this transition was relatively minor.

#### 3.2.3. FEM Analysis

At larger scales, FEM plays a critical role in materials science by complementing experiments to predict mechanical behavior, stress distribution, and thermal stability. For instance, FEM has been applied to hydroxyethyl cellulose films reinforced with activated carbon (AC) to validate experimental trends and guide material optimization, thereby improving efficiency and predictive accuracy [108]. FEM becomes particularly valuable for porous composites, where the classical rule of mixtures falls short due to its assumption of perfect interfacial adhesion and its neglect of the impact of voids and porosity. A detailed FEM study using the unit-cell approach on clay platelet/cellulose nanocomposites demonstrated that increased porosity significantly decreases modulus and strength. It also revealed that low clay content causes shear and inclined tensile failure, while high clay content leads to brittle fracture. Incorporating phase-field damage and cohesive zone models further enabled the accurate prediction of crack initiation and propagation, enhancing understanding of the failure mechanisms [109].

Moreover, defects in fibrous materials significantly impact their mechanical properties. However, the interaction between defects and fiber behavior, especially in cellulosic systems, remains largely underexplored. Richely et al. [110] addressed this gap by integrating synchrotron X-ray diffraction with FEM to study cellulose ultrastructure under tension. Their findings showed that structural defects influence microfibril reorientation during loading, with greater realignment associated with higher initial angles and defect densities. FEM helped quantify the stiffening effect resulting from this reorientation, explaining how defects impact overall mechanical performance. Building on these insights, Drouiche et al. [111] conducted an experimental and FEM-based investigation into the chemical, thermal, and mechanical properties of Doum Palm Leaf Stalk Fiber (DPLSF), composed primarily of cellulose, hemicellulose, and lignin. FEM simulations validated the experimental results while providing further insights into stress distribution and failure mechanisms under tensile loading, particularly after fiber treatment with sodium bicarbonate.

Given the central role of natural fibers such as cellulose in composite reinforcement, FEM has also been widely used to assess their mechanical contribution. For example, Ichu and Cabuya fibers were modeled under quasi-static loading to evaluate stiffness and elastic constants. Results indicated that mechanical performance is strongly influenced by microstructural parameters, such as the crystallinity index and microfibril angle. Longitudinal stiffness was significantly higher than transverse stiffness, and fibers with lower microfibril angles showed superior tensile properties. FEM also revealed non-uniform stress distributions under off-axis loading, where failure occurred more readily due to lower transverse stiffness and weaker interfibrillar bonding [112].

#### 3.2.4. ML Integration

**Figure 10:**
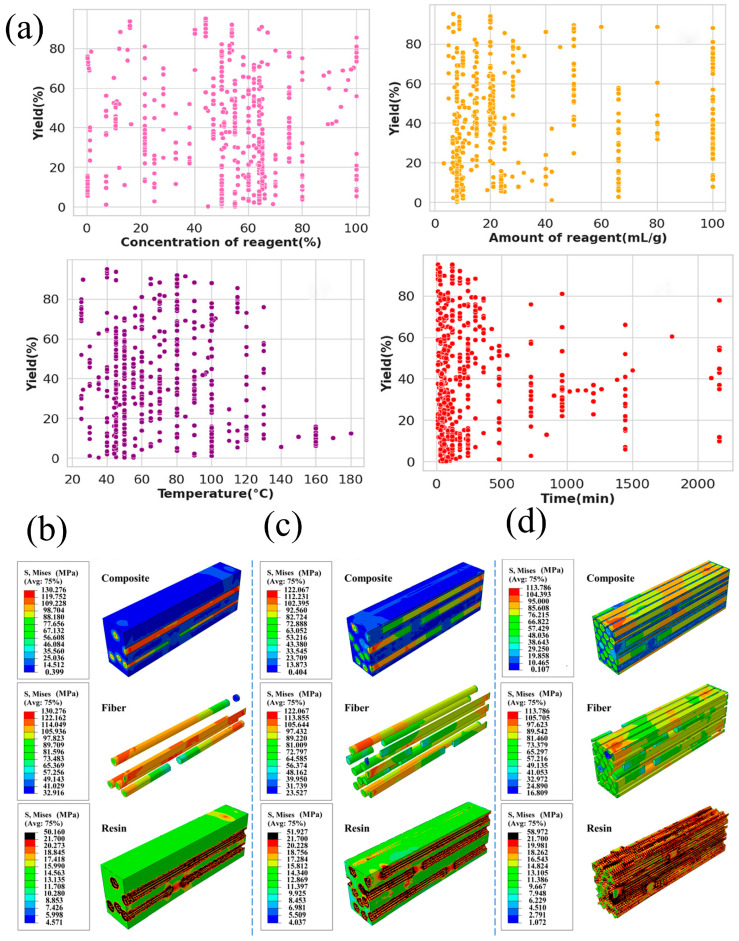
(**a**) Decision tree model illustrating degradation behavior, where color represents degradation rates: fast (yellow), medium (green), and slow (purple) [115,117]. Stress distributions in bamboo fiber–palm oil resin composites with fiber volume fractions of (**b**) 2.6%, (**c**) 28.3%, and (**d**) 63.6%, modeled under a combined interfacial property mode [124], reproduced under the terms of the CC BY 4.0 license.

Following the advances achieved through AAMD, CGMD, and FEM, ML offers a data-driven perspective that complements these traditional simulation techniques. ML has proven to be effective in process optimization and parameter investigation (Figure 10a). In CNC production via sulfuric acid hydrolysis, ML-based models revealed that acid concentration most strongly affects yield, while cellulose source governs crystallinity [113]. When expanded to broader extraction methods, Shapley Additive exPlanations (SHAP) identified optimal reagent concentrations (40–60%) and temperatures (~90 °C) for maximizing CNC yield, while excessive reaction time led to degradation [114,115].

Supervised learning, the most widely used ML approach in materials research, has demonstrated strong potential in predicting the properties of cellulosic materials and their composites [116,117,118,119]. For instance, Anwar et al. [120] developed a predictive model for compressive strength in cellulose nanofiber (CNF)-reinforced concrete, identifying cement and water content as dominant factors through engineering data, sensitivity analysis, and model training. The choice of biomass fibers also plays a critical role in composite performance. Nakayama et al. [121] applied FTIR spectroscopy and specific surface area (SSA) analysis to extract material descriptors, which were used to train regression models capable of predicting the Izod impact energy of lignocellulosic fillers, thereby streamlining the selection of high-performance reinforcements.

Furthermore, ML has advanced the prediction of SSA, a key morphological parameter. Nakayama et al. [122] trained XGBoost and convolutional neural network (CNN) models using sedimentation speed data and heatmap images, achieving an R^2^ of 0.94. This surpassed conventional BET measurements, enabling rapid SSA estimation. Integrating these predictions with FTIR data allowed assessment of PP/CNF composite strength in a highly efficient manner. Importantly, ML is most impactful when integrated into multi-modal pipelines [123]. In a recent study on bamboo fiber–palm oil resin composites, FEM simulations quantified the effects of resin type, fiber volume fraction, and interfacial properties, key parameters informed by SSA. These simulation outputs were then used to train ML models, which identified fiber volume and resin properties as primary predictors of tensile strength, as illustrated in Figure 10b–d [124].

In cases of data scarcity, GANs are the most promising techniques for augmenting data and enhancing performance. Ali et al. employed a GAN to augment spectral data from Near-Infrared (NIR) non-destructive analysis of wood stiffness. Their results revealed that data augmentation significantly improved the model performance with an increase of up to 7.02% in $R^{2}$ and a reduction of 4.29% in root mean squared error [125]. Similarly, Liu et al. employed Wasserstein GAN to augment NI spectra and reported an increase in tree-based ensemble models on predicting mechanical properties [126].

### 3.3. Thermal Properties

#### 3.3.1. AAMD Simulations

**Figure 11:**
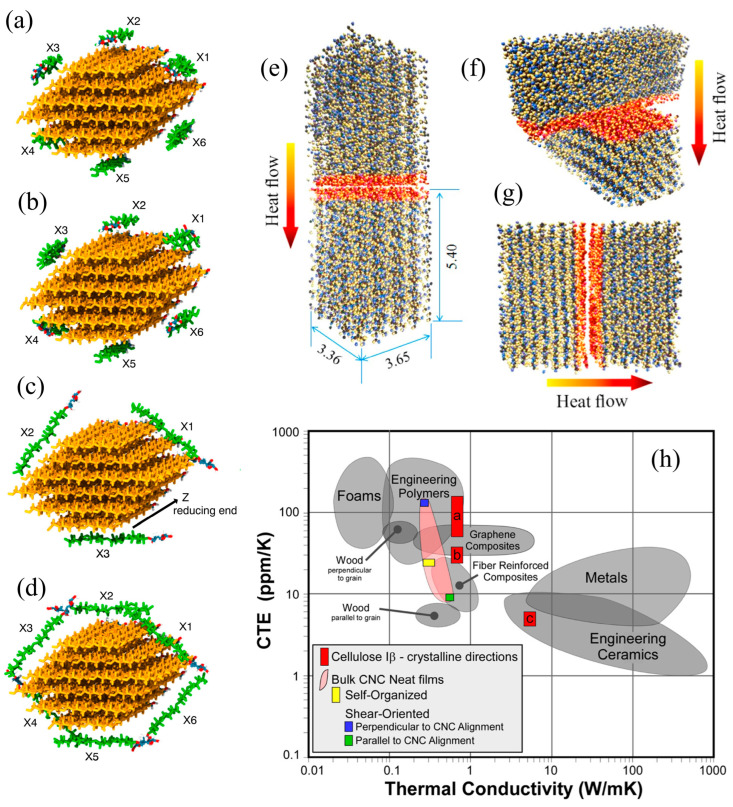
Initial configurations for MD simulations showing xylan chain orientations around cellulose: (**a**,**b**) pre-aligned arrangements and (**c**,**d**) perpendicular arrangements [127], reproduced under the terms of the Creative Commons Attribution (CC BY 4.0) license. Atomistic configurations used to study interfacial thermal resistance in CNCs: (**e**) chain-aligned, (**f**) transverse-perpendicular, and (**g**) transverse-parallel orientations. (**h**) Ashby plot of thermal response of cellulose Iβ and bulk CNC films with respect to other engineering materials. Results for cellulose Iβ and bulk CNC films were based on the MD simulations and experimental measurements, respectively [80], reprinted from, Copyright 2020 American Chemical Society.

MD simulations have revolutionized the analysis of thermal properties by offering atomistic insights that are often inaccessible through experimental techniques. These simulations enable precise modeling and prediction of material behavior under varying temperature conditions, supporting the investigation of fundamental phenomena such as thermal expansion, heat conduction, phase transitions, and molecular mobility (Figure 11a–d) [127]. This capability is particularly valuable for complex polymeric and biomaterials, where thermal behavior is governed by intricate intermolecular interactions, hydrogen bonding, and structural rearrangements.

For cellulose-based materials, MD simulations have provided a deeper understanding of thermal behavior, glass transition temperature (*T_g_*), and the temperature-dependent mechanical properties, such as elastic modulus. These insights are crucial for designing sustainable bio-based thermoplastics with improved processability and performance. A notable example is the work by Elf et al. [80], who used fully atomistic MD simulations to investigate the impact of ring-opening modifications in dialcohol cellulose under both dry and moist conditions. By simulating amorphous cellulose systems at ambient and elevated temperatures (150 °C), and with varying water content (0% and 25%), they observed that ring opening led to significantly increased molecular mobility. This resulted in a reduction in *T_g_*, stiffness, and strength, which is advantageous for improving thermoplastic processability. Furthermore, the simulations showed that increased water content enhanced polymer diffusivity and ductility, highlighting the complex interplay between molecular structure and environmental conditions.

MD simulations have also been applied to understand adhesion in wood–epoxy bonded systems [128]. Temperature-dependent analyses revealed conformational changes in both the wood cell walls and the epoxy, explaining the observed macroscopic weakening of interfacial adhesion at elevated temperatures. Specifically, delamination became more pronounced in the wood component, while the epoxy phase transitioned into a weaker, thin-film structure, reducing both fracture energy and adhesion strength.

To achieve a multiscale understanding of thermal conductivity in CNCs, MD simulations can be effectively integrated with experimental data. Simulations of single cellulose Iβ crystals revealed highly anisotropic thermal conductivity: approximately 5.7 W/(m·K) along the chain direction and 0.72 W/(m·K) transversely (Figure 11e–h) [129]. The phonon mean free path was found to exceed the lateral dimensions of CNCs, suggesting that interfaces play a dominant role in thermal transport. Interfacial thermal resistance was estimated to be low (9.4–12.6 m^2^K/GW), attributed to strong intermolecular interactions, including van der Waals forces and hydrogen bonding. Moreover, heat transfer improved with CNC alignment, as shear-aligned films delivered up to a twofold increase in thermal conductivity compared to isotropic configurations.

#### 3.3.2. CGMD Simulations

**Figure 12:**
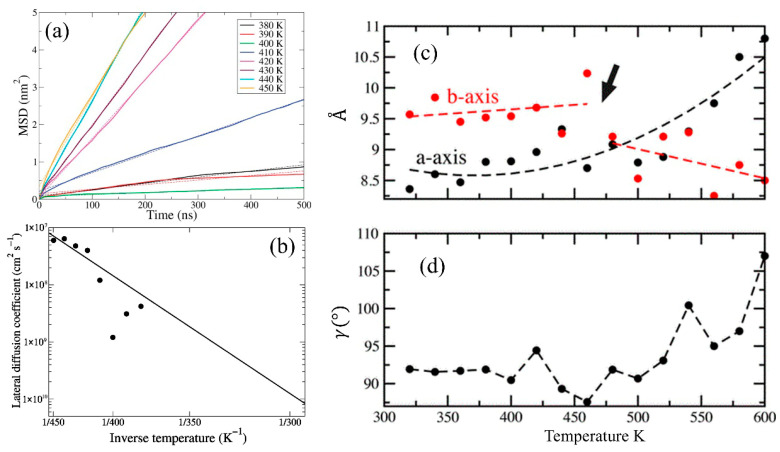
(**a**) Calculated lateral mean square displacements of the CBD, with linear fits used to determine diffusion coefficients (Dl) [68], (**b**) Arrhenius plot of log(Dl) versus inverse temperature (1/T), with Dl calculated from the MSD data at various temperatures [68]. Reprinted with permission from Wohlert et al., A Coarse-Grained Model for Molecular Dynamics Simulations of Native Cellulose. Copyright 2011 American Chemical Society. Temperature-dependent changes in the unit cell parameters of cellulose Iβ: (**c**) a and b axes and (**d**) the monoclinic angle γ. The arrow marks the crystalline phase transition point near 500 K [98], Copyright 2015 American Chemical Society.

The Martini CG model has been employed to study crystalline cellulose microfibers at the mesoscale, revealing their thermal expansion and mechanical responses to bending [98]. These models successfully captured the structural transitions and degradation of cellulose fibers at elevated temperatures, along with their elastic behavior. Wohlert et al. [68] developed a Martini-based CG model for crystalline cellulose to investigate its thermodynamic behavior, including solvent interactions and temperature-dependent diffusion. They parameterized the potential to reproduce partitioning free energies of cello oligomers between water and cyclohexane, effectively capturing the balance between polar and apolar interactions that govern thermal properties. The diffusion of the carbohydrate-binding domain (CBD) on the surface of cellulose was simulated at various temperatures to validate thermal behavior of the model (Figure 12a). According to the lateral mean square displacement (MSD) data, they extracted diffusion coefficients (Figure 12b), conducted Arrhenius analysis, and estimated an energy barrier (ΔG) of approximately ~48 kJ/mol for CBD motion. By applying a time-scaling correction factor (4 × acceleration) based on Martini dynamics, they obtained a diffusion coefficient of 3.1 × 10^−11^ cm^2^/s at room temperature, in good agreement with experimental data. Overall, their method highlights the potential of CGMD in predicting cellulose-related thermal transport and interfacial mobility, which are essential aspects for accurately modeling processing conditions and thermal behavior in cellulose systems.

CGMD also holds great potential for semi-quantitative predictions of thermal properties and phase transformation behaviors in cellulose microstructures, which is critical for understanding thermal processing and material stability in bio-based applications. In one study, Gnanakaran et al. [98] used a Martini-based CG model to investigate the thermal and structural behavior of crystalline cellulose microfibers, focusing on cellulose Iβ and cellulose III_I_ allomorphs. Their model successfully captured temperature-dependent unit cell changes, demonstrating anisotropic thermal expansion behavior (Figure 12c,d). Simulations conducted from 320 K to 600 K showed that while the *a*-axis expanded with increasing temperature, the *b*-axis contracted beyond 500 K, indicating a phase transformation into an amorphous structure, a result consistent with experimental decomposition temperatures. Notably, the model reproduced experimental thermal expansion coefficients with acceptable accuracy (e.g., −4.5 × 10^−4^ °C^−1^ at high temperature). Simulations of a hundred-mer microfiber revealed that the thermal destabilization mainly influenced the outer fiber layers, while the core retained crystalline order up to ~500 K.

Furthermore, CG-based models can capture temperature-dependent bond dynamics and reveal the plasticity of hydrogen-bond networks in cellulosic materials under thermal stress, which is critical for understanding biomass pretreatment. Shen et al. [130] employed a CG model to investigate the thermal stability of cellulose based on hydrogen-bond networks. Their analysis focused on two types of hydrogen bonds: intrachain (within a single cellulose chain) and interchain (between different chains). They found that intrachain hydrogen bonds are more thermally stable than interchain ones, with the latter breaking around 500 K. Their model also demonstrated that different bond patterns emerged at different temperatures, suggesting that cellulose undergoes gradual structural reorganization rather than abrupt failure when exposed to heat.

#### 3.3.3. FEM Analysis

FEM, when coupled with experimental data, provides valuable insights into the thermal stability and mechanical performance of natural fibers, such as degummed Doum Palm Leaf Stalk Fiber (DPLSF). Drouiche et al. [111] utilized ABAQUS simulations to reproduce and interpret the experimentally observed thermal behavior and structural evolution of the DPLSF after sodium bicarbonate treatment. The simulations confirmed a shift in thermal degradation temperature from 290 °C to 330 °C post-treatment, demonstrating enhanced thermal stability and highlighting FEM as an effective tool for evaluating and optimizing the thermal and mechanical properties of cellulosic materials.

Incorporating FEM with advanced characterization methods, such as micro-computed tomography (μCT), can yield even deeper insights. Qiu [131] demonstrated the utility of combining FEM with μCT to simulate mesoscale heat conduction in wood. Utilizing actual microstructural inputs, the study revealed the directional dependence of thermal conductivity, with enhanced heat flow along the grain axis. The model also showed significant influences of porosity and moisture content on thermal behavior, providing a high-resolution, non-invasive strategy for studying complex, heterogeneous wood structures.

Further advancements were presented by Díaz et al. [132], who employed a computational homogenization-based multiscale simulation framework to analyze the thermal performance of cross-laminated timber (CLT). Their approach began at the nanoscale, accounting for the contributions of cellulose, hemicellulose, and lignin, and advanced to the microscale by incorporating detailed cell wall structure. FEM was applied at each level to homogenize thermal properties and derive effective thermal conductivity values. Their findings confirmed the highly anisotropic nature of wooden materials and demonstrated that thermal behavior is strongly influenced by the cellulose volume fraction and microfibril angle. Moreover, thermal dissipation across CLT panels was examined, where different joint configurations were evaluated. The study concluded that U-type joints offer better insulation performance than Z-type joints, emphasizing the importance of structural design in thermal regulation.

Additionally, the Finite Volume Method (FVM) has been used to explore the spatial variability of the Representative Elementary Volume (REV) across different wood species, addressing challenges often encountered by traditional methods [133]. In a comparative study involving broom grass, fishtail palm, and sansevieria fiber-based composites, FVM analysis showed that increasing fiber volume fraction enhanced thermal insulation. Notably, fishtail palm composites achieved the lowest thermal conductivity at maximum fiber content, measuring only 0.0163 W/m·K. All three materials demonstrated strong insulation performance, making them promising candidates for energy-efficient and sustainable applications [134].

#### 3.3.4. ML Modeling

**Figure 13:**
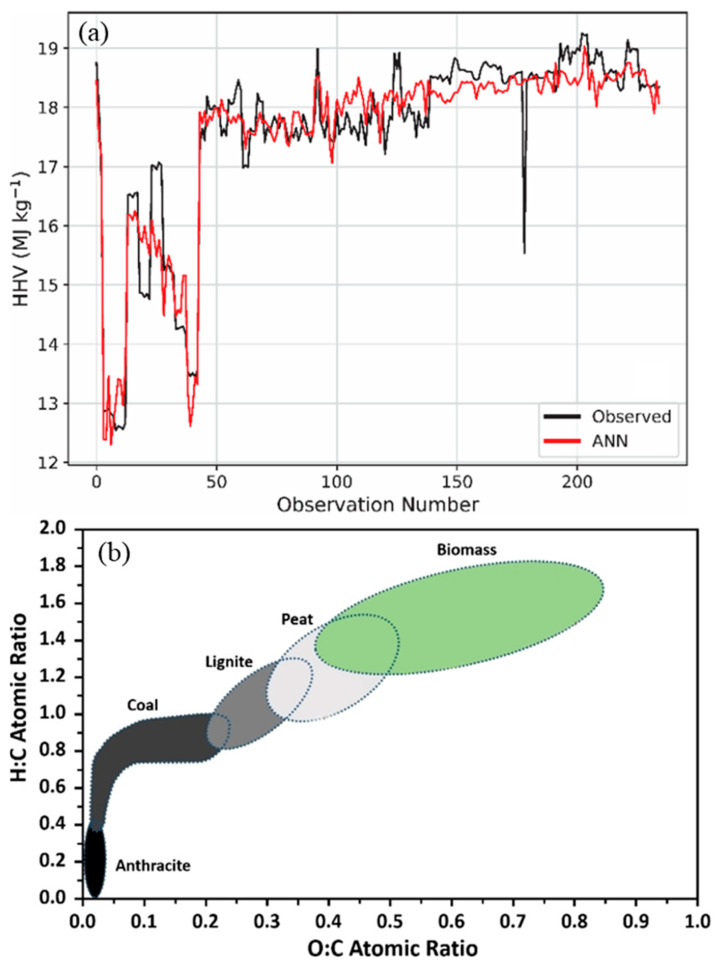
(**a**) ANN estimation of the HHV of biomass across different observation numbers [135], reproduced under the terms of the CC BY 4.0 license. (**b**) Van Krevelen diagram illustrating the hydrogen-to-carbon versus oxygen-to-carbon ratios for various samples [139], reproduced under the terms of the CC BY 4.0 license.

ML has emerged as a powerful tool for modeling and predicting various aspects of thermal behaviors. One such application involves predicting the higher heating value (HHV) of biomass based on its structural constituents: cellulose, hemicellulose, and lignin. ANNs were trained and optimized for this task, followed by global sensitivity analysis to identify key influencing factors. Results indicated that lignin and hemicellulose positively contribute to HHV, while cellulose exhibits a negative correlation (Figure 13a) [135].

Beyond HHV, heat capacity plays a critical role in the thermal performance of cellulosic biomass. Traditional methods for measuring heat capacity across a wide temperature range can be costly and time-consuming. ML models offer a rapid alternative, enabling accurate predictions under diverse conditions. The results revealed that temperature and ash content positively influence heat capacity, whereas crystallinity and sulfur content exhibit inverse effects [136].

ML techniques have also been employed to explore thermal degradation behavior of vegetal fibers using compositional data derived from thermogravimetric analysis (TGA) [114]. Simulated TGA curves demonstrated that hemicellulose degrades at the lowest temperatures, lignin leaves the highest residual mass, and cellulose shows minimal residue at high temperatures. Subsequently, ANN models integrated with a Sequential Regression Method (SRM) were used to predict the thermal response of various fiber compositions. These models revealed that thermal stability improves with higher lignin and cellulose content, while hemicellulose accelerates early degradation, establishing a robust framework for optimizing the thermal characteristics of lignocellulosic materials.

Furthermore, differential thermogravimetric (DTG) analysis data were used to train ML models to predict biomass component degradation profiles based on proximate analysis, eliminating the need for experimental TGA tests [137]. The results validated the general thermal behavior: hemicellulose decomposes at lower temperatures, cellulose exhibits rapid breakdown, and lignin remains the most thermally stable component. In scenarios where TGA experiments are impractical, ML can fill the gap by leveraging existing combustion data. For example, Sezer et al. [138] developed an ML model optimized using a Bayesian regularization algorithm to predict the biomass combustion index, which is a crucial parameter in the evaluation of combustion performance. This model demonstrated high accuracy and eliminated the need for time-consuming experimental procedures, and completely aligned with the Van Krevelen diagram illustrated in Figure 13b, which means all predictions were related to the biomass area. Moreover, the practical effectiveness of the ML model (RF) in predicting activation energy (Eα) during biomass pyrolysis for three unexploited biomass types (peanut shell, ramie stick, and sawdust) with a strong agreement between model predictions and experimental results for different conversion degrees (α) confirms the potential of ML in this area.

### 3.4. Electronic Properties

Cellulosic materials are primarily recognized as durable and strong bio-based candidates for various applications. Beyond their mechanical and thermal properties, cellulosic materials are increasingly being explored for electronic and dielectric applications [140]. Their inherent electronic characteristics are gaining attention for novel uses in bioelectronics, flexible electronics, and sustainable energy devices [141,142]. Therefore, understanding the factors that influence the electronic properties of cellulosic materials is crucial for effectively harnessing them for specific technological applications and accelerating the design and modification of electronic devices with tailored performance.

Computational techniques, particularly simulations, have emerged as powerful tools for advancing this understanding, offering detailed insights into the underlying mechanisms and principles. Among these, DFT provides valuable information regarding the electronic structure, band gap, and charge transport mechanisms, enabling the prediction of the electronic properties as the molecular structure is tuned [143]. For instance, Bi et al. [144] employed DFT to investigate the electronic interactions between cellulose, carboxymethyl cellulose, and lignin in the development of transparent cellulosic materials. Using the B3LYP/6-311G method, they calculated the electrostatic potential (ESP) map and surface charge distributions for each component. Their results demonstrated that the carboxymethyl cellulose exhibits a larger polar surface area and a more negative average ESP compared to untreated cellulose, suggesting enhanced electrostatic repulsion and improved dispersion of lignin molecules. The study showcased how DFT can be applied to predict and optimize molecular interactions in bio-based optical materials. The resulting materials exhibited high optical transparency, strong UVA resistance, and hydrophobicity, making them promising eco-friendly alternatives to plastic for packaging applications.

Although cellulose is traditionally considered an insulating biopolymer, its electronic behavior can be tuned through chemical modifications, structural adjustments, or the incorporation of conductive elements. Srivastava et al. [145] conducted a comprehensive DFT-based investigation into the electronic properties of various crystalline cellulose allomorphs (I_α_, I_β_, II, and III_1_) as well as chemically modified cellulose structures. They employed periodic DFT calculations using the PBE-D2 functional and triple-ζ-valence basis sets to optimize the crystal structures, followed by calculations of full electronic band structures and density of states (DOS). Their findings confirmed that all native cellulose allomorphs are electronic insulators, with predicted band gaps ranging from 5.2 to 5.5 eV using the PBE functional and up to 8.2 eV with the hybrid PBE0 functional. While some dispersion was observed near the valence and conduction band edges, the bands were relatively flat, indicating highly localized electronic states. In addition, they modeled the chemical modification of cellulose Iβ via amidation with aniline and 4,4′-diaminoazobenzene to explore the tunability of cellulose electronic properties. The modification introduced new electronic states within the band gap, narrowing the effective gap to 4.0 eV and 1.8 eV, respectively. The mid-gap states, primarily arising from the aromatic group of the modifiers, suggest that functionalized celluloses may absorb in the visible spectrum, making them suitable for photonic or optoelectronic applications. Moreover, they computed vibrational spectra (IR and Raman) to support structural characterization and correlate with experimental data. By integrating structural, vibrational, and electronic analyses, their work demonstrated the utility of DFT in not only explaining the inherent insulating nature of cellulose but also in predicting how surface modifications can be used to tailor its electronic band structure. These findings establish cellulose as a promising candidate for sustainable electronic and photonic materials.

To accurately predict the relative energies and geometries of hydroxyl-containing compounds, the B3LYP functional with diffuse and polarization corrections was utilized. Grimme’s methods, including DFT-D1, D2, D3, and D4, are highly effective approaches for simplifying van der Waals (vdW) or dispersion interactions. These methods involve the construction of simple pairwise force fields, which are specifically tuned for widely used DFT functionals, such as PBE and B3LYP. Among them, the PBE-D2 method (Perdew-Burke-Ernzerhof with D2 dispersion correction) demonstrated superior predictive ability compared to other methods, including the vdW-DF (van der Waals Density Functional). Researchers believe that the well-resolved structure and prevalence of cellulose Iβ enable more reliable DFT calculations, providing profound insights into the molecular and electrostatic properties of this vital biopolymer.

In lignin-carbohydrate complexes, the C–O bond attributes are largely strongly influenced by adjacent aliphatic, aromatic, or carbonyl groups and show considerable variation. C–O bond enthalpies are significantly higher when associated with aromatic moieties, highlighting electron delocalization near the aromatic ring [146]. Quantum mechanical (QM) calculations and MD simulations revealed strong H-bonding interactions, for example, TsOH/pentanol with VG: −2460 kJ/mol, AlCl3/MTHF with DX: −2385 kJ/mol, which play key roles in enhancing the efficiency of xylan and lignin removal. These interactions were found to be crucial for improving removal efficiency in biphasic pretreatment processes [147]. Electron charge density mapping further indicated that cellulose-hemicellulose pairs share more hydrogen bonding than the cellulose-lignin monomer pairs [32].

Understanding the frequency-dependent dielectric behavior of crystalline cellulose is essential for advancing its applications in sustainable electronics and photonic materials. To this end, Yadav et al. [148] employed DFT with dispersion corrections to explore the dielectric and optical behavior of cellulose Iα and Iβ. They utilized the Quantum ESPRESSO package and implemented different exchange-correlation functionals (LDA, GGA, PBE-D3), applying scissor corrections to adjust for the underestimation of the electronic band gap, thereby enabling more accurate optical properties. They calculated both the real and imaginary parts of the dielectric function and revealed clear anisotropy along the *xx*, *yy*, and *zz* directions. Variations in hydrogen bonding and crystal packing led to distinct optical responses between cellulose Iα and Iβ. The study also demonstrated good agreement between theoretical and experimental values after applying the electronic band gap corrections.

**Figure 14:**
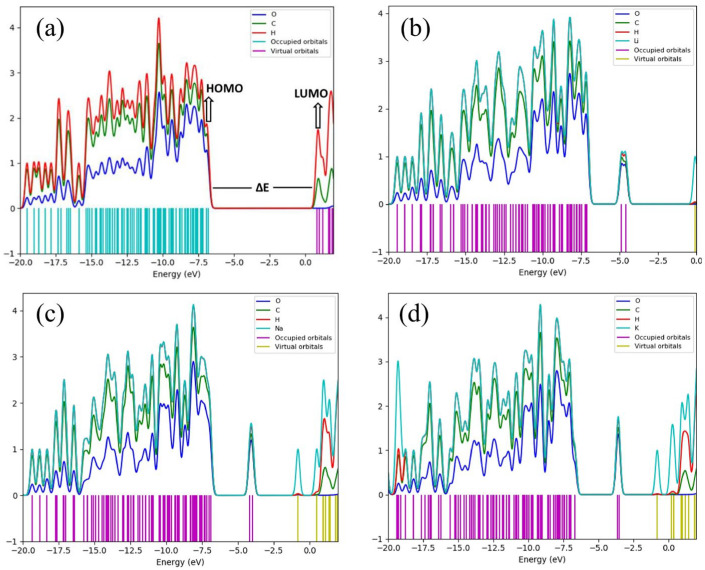
PDOS plots of (**a**) pristine cellulose, (**b**) cellulose-1Li, (**c**) cellulose-1Na, and (**d**) Cellulose-1K [149], reprinted under a CC BY 4.0 license.

Recent studies have shown that alkali modification can tailor cellulose to enhance its optoelectronic performance. Refaat et al. [149] conducted a DFT investigation into the effects of alkali metal dopant inclusion in cellulose and its impact on electrostatic and spectroscopic behavior (Figure 14a–d). They applied the B3LYP/6-311G(d,p) level of theory to compute pristine and alkali-modified cellulose chains, studying changes in molecular orbital levels, band gaps, and electrostatic potential distributions. Their findings revealed significant reductions in the HOMO–LUMO band gap due to the incorporation of alkali metals, from 4.89 eV in pristine cellulose to as low as 2.47 eV in hydrated K-doped cellulose, indicating increased electronic activity. Projected density of states (PDOS) analysis revealed new orbital contributions near the Fermi level from the metal atoms, along with enhanced charge delocalization across the energy spectrum involving both metal and carbon atoms.

To understand the role of cellulose degradation products, Kumar et al. [150] conducted an in-depth theoretical study on the electronic and optoelectronic properties of representative chromophores formed from thermal and oxidative degradation of bacterial cellulose (BC): 2,5-dihydroxy-[1,4]benzoquinone (A), 5,8-dihydroxy-[1,4]naphthoquinone (B), and 2,5-dihydroxyacetophenone (C). using DFT (B3LYP and PBE) and time-dependent DFT (TDDFT) with 6-31G* and 6-31+G* basis sets, they computed ionization potentials, electron affinities, quasiparticle energy gaps, and optical absorption spectra in vacuum. Molecule B exhibited a clear onset of optical excitation in the visible range and had the smallest quasiparticle gap. They also assessed exciton binding energies, which were particularly strong in Molecule B, up to 2.6 eV, indicating tightly bound electrons.

Interestingly, they extended the study using classical MD simulations to obtain solvated conformations of each chromophore in water, followed by TDDFT calculations to study solution-phase absorption spectra to get the impact of medium effects. The calculations revealed solvation-induced redshifts in absorption peaks and lowered exciton binding energies, highlighting the significant influence of the solvent environment on chromophore behavior.

### 3.5. Catalytic Properties

The application of the catalytic properties of cellulose is primarily associated with its interaction with enzymes and chemical catalysts during hydrolysis and the transformation of cellulose into bioactive molecules. Recent advancements in computational techniques have enabled researchers to gain a molecular-level understanding of how cellulose degrades via enzymatic mechanisms involving cellobiohydrolases [151], endoglucanases [152], and β-glucosidases [153]. These developments highlight the importance of MD and DFT in elucidating catalytic behavior [154,155]. The applications of these methods are discussed below.

The use of ionic liquids (ILs) for cellulose depolymerization has emerged as a promising avenue due to their ability to disrupt hydrogen-bond networks and catalyze selective chemical conversions [82]. Computational methods, including MD and DFT, have played a critical role in resolving the mechanisms of cellulose-ILs interactions and enabling the rational design of catalytically active IL systems.

In a combined theoretical and experimental study, Kumar et al. [156] developed a series of Brønsted acidic ionic liquids with conjugated cations and demonstrated their effectiveness in converting cellulose microcrystals into 5-hydroxymethylfurfural (5-HMF) and levulinic acid under biphasic conditions. Using DFT and natural bond orbitals (NBO) analysis, they investigated the electronic structures and interaction energies of IL ion pairs. The results showed that increased electron delocalization and higher proton affinity were directly correlated directly with increased catalytic activity. Under optimal conditions, they achieved a 70% yield of 5-HMF, highlighting the value of theory-guided IL design for biomass valorization.

Complementing this work, Liu et al. [82] employed classical MD simulations to explore the dissolution mechanism of cellulose in [C_2_mim][OAc], a widely used IL in biomass pretreatment. Their findings revealed that acetate ions form strong hydrogen bonds with cellulose hydroxyl groups, thereby destabilizing the crystalline structure and improving access to catalytic sites. The simulation also showed that cellulose undergoes structural transitions in the presence of the IL, potentially increasing the percentage of catalytically active glycosidic linkages. Their results provided molecular-level evidence for the enhanced solvation and catalytic activity observed with acetate-based ILs.

**Figure 15:**
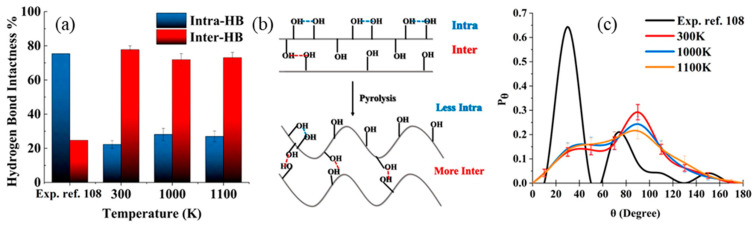
(**a**) Intrachain and interchain hydrogen bond (HB) distributions of cellulose at various pyrolysis temperatures. (**b**) Schematic representation illustrating the transformation of the intra- and interchain HB networks with increasing pyrolysis temperature. (**c**) Angular distribution of HBs, determined by analyzing the angle between each HB and the positive Z-axis, reprinted with permission from [157], copyright 2020 American Chemical Society.

Furthermore, Qiao et al. [157] utilized reactive MD (ReaxFF) simulations to investigate the pyrolytic decomposition of crystalline cellulose under thermal treatment. Their study revealed how thermal activation disrupts the hydrogen-bond network, rearranges carbon–hydrogen–oxygen species, and reduces mechanical strength (Figure 15a–c). The simulations captured the formation of critical intermediates, such as levoglucosan and furanic structures, mirroring the reaction pathways observed in IL-based systems, as studied by Kumar et al. [156]. These insights highlight how integrated experimental and modeling approaches can inform the design of more efficient, IL-based catalytic systems tailored for mild and sustainable biomass deconstruction.

ReaxFF is particularly effective for modeling the catalytic behavior of cellulose, as demonstrated by Feng et al. [158], who employed it to investigate the combustion mechanisms and kinetics of active cellulose. Their study detailed the reaction pathways and highlighted the formation of key products, such as CO_2_, H_2_O, and hazardous intermediates including CO and formaldehyde, which are mainly driven by free-radical reactions. Kinetic analysis further revealed that cellulose combustion follows first-order kinetics, with an activation energy of 114.42 kJ/mol.

Although chemical and ionic liquid-based approaches provide effective tools for catalyzing cellulose, enzymatic hydrolysis remains the most selective and sustainable technique for converting cellulose into fermentable sugars. The efficiency of this process relies heavily on the activity of enzymes, such as cellobiohydrolase Cel7A, which cleaves cellulose chains from their termini.

Computational and structural studies in recent years have deepened our molecular-level understanding of what happens when Cel7A interacts with cellulose, advancing insights into enzyme–substrate binding, processivity, and catalytic turnover. Yang et al. [159] employed replica-exchange MD simulations to study the insertion of a cellulose chain into the catalytic tunnel of TrCel7A, the cellobiohydrolase from *Trichoderma reesei*. Their simulations demonstrated that chain insertion is entropically driven and facilitated by hydrophobic interactions with aromatic residues lining the tunnel. They further proposed that energy released during glycosidic bond cleavage contributes to pulling the cellulose chain through the enzyme, offering a mechanistic explanation for the processivity of Cel7A.

Building on these findings, Textor et al. [151] integrated X-ray crystallography and classical MD simulations to compare the catalytic core domain of *T. harzianum* Cel7A with that of *T. reesei*. Structural comparison revealed key differences: *T. harzianum* exhibited a shorter loop at the tunnel entrance and greater flexibility in Tyr260, a residue involved in chain interaction. These structural features correlated with improved enzyme kinetics (higher *k_cat_* and lower *K_m_*), suggesting that subtle changes in tunnel architecture and dynamics can significantly enhance catalytic efficiency. In a follow-up study, Yang et al. [159] conducted long-timescale MD simulations to analyze residue-level contacts between TrCel7A and the cellulose chain at various tunnel positions. Their results identified residues, such as E212, D214, and E217, as critical for maintaining productive binding and catalysis. Additionally, the study mapped changes in contact frequency during chain translocation, reinforcing the importance of aromatic residues in guiding the substrate and stabilizing intermediate states.

Beyond enzymatic and ionic liquid-catalyzed pathways, thermal decomposition and alkaline hydrolysis represent essential catalytic routes for cellulose deconstruction. A deep understanding of these processes at the atomic level is vital for optimizing pretreatment conditions and developing efficient thermochemical biomass valorization methods. Using DFT and ReaxFF, recent studies have yielded valuable mechanistic insights into how temperature, base catalysts, and oxidative environments affect glycosidic bond scission and overall cellulose structure [160,161]. In a fundamental study, Mao et al. [162] used DFT to investigate the mechanism of alkaline thermal hydrolysis in cellobiose, a disaccharide model for cellulose. Their analysis revealed a two-step reaction pathway: (1) proton abstraction by a hydroxide ion and (2) glycosidic bond cleavage leading to glucose residue formation. The first step, with an energy barrier of 147.11 kJ/mol, was identified as the rate-limiting step. Using ETS-NOCV analysis, they quantified electron transfers and visualized orbital interactions, which are essential for understanding bond cleavage. Thermodynamic and kinetic simulations further showed that increasing temperature enhances both the feasibility and rate of hydrolysis, supporting improved catalytic performance under elevated thermal conditions.

Similarly, Qiao et al. [157] employed ReaxFF MD simulations to explore the thermal decomposition of crystalline cellulose under pyrolytic conditions. Their simulations captured real-time bond-breaking and formation events, offering insights into the disruption of hydrogen-bond networks, atomic rearrangements, and the formation of volatile compounds, such as levoglucosan.

## 4. Summary and Conclusions

This review has outlined the most common simulation and computational techniques used for cellulosic materials, including DFT, MD, FEM, and ML. As discussed and illustrated throughout, we aimed to highlight the applications spanning the atomic to mesoscale in a comprehensive manner.

Recent developments in these methods and their utilization in biopolymer investigations have revealed valuable insights into the hierarchical structure and microstructure of cellulose, hemicellulose, and lignin. These techniques have enabled a deeper understanding of the structural, mechanical, thermal, electronic, and catalytic properties of biopolymers, particularly those derived from cellulosic materials. Furthermore, the integration of ML with other multiscale simulation techniques allows researchers to investigate and predict material behavior under previously unexplored conditions. The employment of such methods fosters our understanding of atomic- and molecular-level interactions, offering profound insights into the fundamental nature of these materials.

Sophisticated methods, such as DFT calculations, have unraveled the intricate molecular structures involved in lignin and xylan removal. Additionally, AAMD simulations have contributed invaluable insights into the mechanical behavior of cellulose, hemicellulose, and lignin. AAMD has also been successfully applied to study thermal properties, revealing how these materials behave under varying temperatures. Moreover, CGMD enables the exploration of mechanical and thermal properties across different structural levels, aligning simulation outputs closely with experimental results. CGMD also provides a means to assess the impact of thermal energy on the stability and integrity of biopolymers, which is crucial for understanding their performance in diverse environmental conditions.

On a larger scale, FEM has been employed to model mechanical and thermal responses under diverse conditions, such as varying temperature or moisture levels. While FEM has been applied to investigate a wide range of properties, its most common applications remain in the areas of thermal and mechanical analysis, due to the versatility of its analytical and computational frameworks. The emergence and integration of ML have further revolutionized the application of computational modeling in this field. Various ML models, such as ANN and CNN, have been effectively used to model and predict the behavior and properties of cellulosic materials. These models have demonstrated high efficiency and predictive accuracy across various domains, including paper manufacturing, materials fabrication, and biomass processing. These advancements collectively highlight the transformative potential of computational modeling to not only enhance our understanding of cellulosic materials but also to drive innovation in renewable nanomaterials, fuels, and bio-based chemicals.

Although the multiscale computational methods discussed herein provide powerful tools for understanding the structure and behavior of cellulose, hemicellulose, and lignin, notable discrepancies remain between simulation and experimental observations. For instance, atomistic MD simulations often predict axial elastic moduli of cellulose Iβ that are substantially higher than experimentally measured values for bulk fibers or cellulose nanocrystal (CNC) films. This deviation is largely attributed to real-world factors, such as structural imperfections, moisture content, fibril misalignment, porosity, and disordered domains, that are typically absent from idealized models. Similarly, MD and CGMD simulations frequently predict highly anisotropic thermal conductivity along the polymer chain axis; however, experimentally measured values are considerably lower, primarily due to interfacial phonon scattering and incomplete fibril alignment. Moreover, many computational studies assume ideal bonding environments or uniform chemical functionalization, whereas natural fiber systems are inherently heterogeneous in structure and chemistry.

These discrepancies underscore the need for improved computational frameworks that more accurately capture the multiscale heterogeneity of cellulose-based materials. Promising directions include hybrid multiscale approaches, where force field parameters are continuously validated against experimental data, and the adoption of digital twin frameworks that integrate real-time experimental feedback to iteratively refine model predictions. In addition, combining high-throughput experimental screening with ML-driven surrogate modeling can enable simulations parameterized by statistically representative material variability rather than idealized unit cells. Future computational models that explicitly link simulation parameters to experimentally measurable descriptors, such as crystallinity index, microfibril angle, water activity, and interfacial hydrogen bond density, will offer greater predictive accuracy and practical relevance for designing high-performance, sustainable cellulosic materials.

Since each technique possesses distinct characteristics, Table 1 is presented to provide a general comparison and a clearer understanding of their respective capabilities. Depending on the targeted scale and application, any of these methods can be employed individually or integrated within a multiscale computational framework.

Despite the strengths of each computational technique, several limitations and challenges remain when applying them to complex material systems. Achieving interpretable multiscale results requires addressing these limitations in accordance with the specific physical phenomena and objectives under investigation. DFT provides highly accurate insights into bonding and electronic structures; however, it is constrained by computational cost and is unsuitable for large or heterogeneous systems such as biopolymers with intricate polymeric networks [163]. MD enables simulations of larger systems and longer timescales, but is limited by the accuracy of classical force fields, which often fail to capture reactive events such as bond breaking or dynamic crosslink exchange commonly observed in biopolymeric systems [164]. FEM effectively models macroscopic mechanical behavior but lacks molecular-level resolution. Moreover, the inherent viscoelasticity and structural complexity of polymeric materials pose additional challenges that FEM cannot fully capture or interpret [165]. ML, while capable of uncovering hidden structure–property relationships in complex systems, is highly dependent on the quality and quantity of available data. The absence of large, standardized, and high-quality datasets constrains the generalizability, interpretability, and validation of ML models, limiting their predictive reliability [166,167].

Although ML has proven beneficial in predicting structural, mechanical, and thermal properties of cellulosic materials, the predicted reliability of the models will strongly depend on transparent dataset construction and validation practices. In most studies, datasets are compiled from a mix of available experimental results in literature and derived features from properties extracted with MD, DFT, or FEM simulations, and generally have a size between tens to a few hundred samples based on the measurement type and cellulose source [115,121,124]. To ensure reproducibility, data provenance must be reported explicitly, by identifying and describing the data source (hand-curated from experimental reports, MD, DFT, or FEM simulations), feature engineering process, and preprocessing methods, such as normalization, scaling, or transformation. Moreover, model validation is particularly crucial in biopolymer systems due to the natural heterogeneity in their characteristics and properties, such as composition, crystallinity, and additional content. The key consideration is employing cross-validation to avoid overfitting, and also using an external hold-out test set to assess generalization ability. Not only validation, but uncertainty quantification is also essential when ML is employed for materials selection. Techniques such as bootstrapped ensemble models, Gaussian Process regression, Bayesian neural networks, and prediction interval estimation can be used to analyze the confidence ranges in target predictions, especially when the dataset is small or derived from diverse experiments with a large amount of noise.

In addition, although there were significant advances in ML-based interatomic potentials recently, such as developing GAP, DeePMD, and NequIP with impressive accelerations in molecular simulations, training high-accuracy force fields for carbohydrates still has severe challenges, including complex hydrogen-bonding networks, conformational flexibility, and stereochemical interactions, which require diverse and high-quality DFT reference configurations [168,169,170]. To develop carbohydrate-focused ML potential successfully, active learning loops are one of the most promising strategies, where new training configurations are added iteratively when model uncertainty is high [171]. Hence, transparent data reporting and compilation, reliable and strict validation methods, uncertainty quantification, and systematic, well-structured methods for ML interatomic potential development are critical parameters for ensuring that ML workflows in cellulosic biopolymer research remain reproducible, interpretable, and physically grounded.

To address these limitations, integrating these techniques enables the strengths of one method to compensate for the weaknesses of another. Establishing an integrated pipeline that incorporates DFT, MD, FEM, or ML enables information to flow and propagate across scales, creating a self-reinforcing loop that enhances both understanding and predictive capabilities.

**Figure 16:**
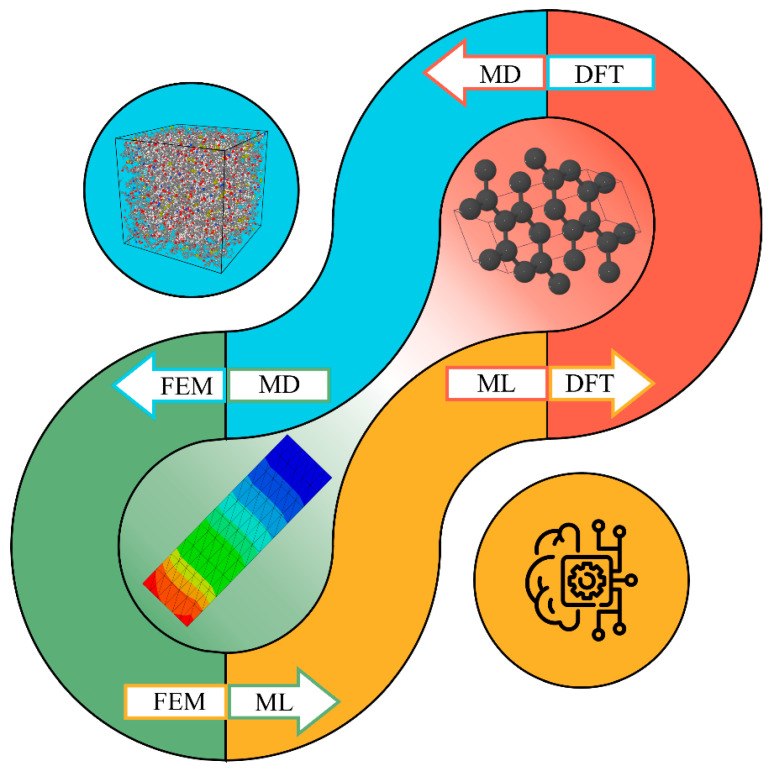
Schematic illustration of the feedback loop integrating four computational techniques.

To clarify how information propagates across scales and how to bridge the gap between atomic-level mechanisms and macroscopic performance, an integrated pipeline can be established to create a learning loop that incorporates DFT, MD, FEM, and ML in a sequential and data-driven manner. In this framework, DFT calculations could be the first technique to be utilized for providing fundamental energetic and structural parameters at the atomic scale. These parameters will then be used as the input in MD simulations to model molecular interactions, dynamic behavior, and temperature-dependent responses. The obtained properties via MD, such as elastic modulus, diffusion coefficients, or relaxation times, are subsequently transferred into FEM models to evaluate the mechanical, thermal, or rheological performance at the component or structural scale. Finally, ML models will be trained on datasets generated across all three computational stages, which identify underlying structure–property relationships and enable a comprehensive fundamental understanding, rapid prediction, and optimization of desired systems. The entire process is illustrated in Figure 16 This hierarchical data propagation pipeline provides a coherent pathway for understanding and designing cellulosic biopolymers across multiple length and time scales.

## 5. Outlook and Future Directions

Looking ahead, the exploration of novel bio-based materials, particularly cellulose and lignin, holds immense potential for innovation. This progress is poised to reshape materials selection and design processes through the strategic application of computational techniques and ML. These approaches provide the opportunity to streamline development timelines, minimize environmental impacts, and promote more sustainable practices across various industries.

One promising direction is the integration of ML with the aforementioned simulation techniques to expand their capability in modeling behavior across broader spatial and temporal scales. Currently, significant gaps exist between computational and experimental results. ML can serve as a bridge by analyzing discrepancies and guiding refinements in models to improve their predictive accuracy.

The integration of MD with continuum-level methods offers another pathway toward achieving a comprehensive, multiscale understanding of materials behavior, ranging from atomic to the macroscopic level. Such hybrid approaches enable detailed analysis of complex phenomena, including atomic and molecular interactions, structural mechanics, and materials performance under diverse environmental and mechanical conditions. Furthermore, ML has the ability to detect hidden patterns and relationships, which can unlock novel insights into the behavior of bio-based materials and significantly accelerate the pace of discovery. Coupled with high-throughput simulation, ML can help identify optimal formulations and processing parameters for emerging green materials.

Computational methods can also be harnessed to assess the environmental impact of bio-based materials across their entire life cycle. By simulating parameters such as resource consumption, energy usage, and waste generation, researchers can predict and improve the sustainability of new materials before they are produced. These insights support the design of natural materials that are not only functionally advanced but also environmentally resilient. From this perspective, researchers are empowered to develop strategies that minimize environmental footprints and advance the core principles of the circular and sustainable economy. In general, the interdisciplinary nature of computational approaches strengthens innovation and accelerates the development of materials with tailored environmental and functional properties.

Looking ahead, the integration of advanced computational techniques with intelligent data-driven systems is expected to transform the research landscape for materials, such as cellulosic biopolymers. A particularly promising direction is the development of digital twins for biomass-derived materials, in which multiscale modeling, ranging from DFT to FEM, is dynamically coupled with experimental feedback in real-time. Such digital twin frameworks can simulate processing conditions, microstructural evolution, and long-term performance, enabling predictive control over key parameters. For example, digital twins could be employed to regulate cellulose fibril alignment or moisture-induced swelling in natural fiber composites, thereby mitigating internal defects, warping, and loss of strength during processing. Moreover, automated systems can rapidly adjust pretreatment chemistry, catalyst concentration, solvent conditions, and cellulose source, while ML models analyze spectral, mechanical, and thermal outputs to identify previously unexplored property spaces with minimal human intervention. Through a rapid, iterative loop of simulation, prediction, experimentation, and model refinement, the development cycle for cellulose-based films, composites, packaging materials, and bio-derived structural components can be substantially accelerated.

Moreover, sustainability is increasingly becoming an essential consideration in material design. Future computational frameworks are expected to integrate environmental performance metrics directly into ML-assisted optimization, encompassing lifecycle assessment (LCA), carbon footprint, recyclability, biodegradability, and circularity indices. Sustainability-oriented AI models could investigate and propose synthesis procedures or material formulations that simultaneously meet desired properties, durability, and environmental performance, while also assisting in decision-making for environmentally desirable alternatives. Lastly, combining generative models, such as VAEs and GANs, with search spaces that are validated via simulation can support inverse design [172], wherein desired mechanical, thermal, and optical properties will serve as the starting point for generating new bio-based polymer compositions [173]. Together, these emerging trajectories may represent transformative shifts in cellulose materials research from characterizing descriptively, toward predictive, scalable, and circular design, and refining pathways for tailored biopolymer systems that are high-performance and low-carbon.

Ultimately, as computational tools continue to evolve, they will play a pivotal role in shaping the future of research into natural and bio-based materials, opening new frontiers for discovery across a wide range of scientific fields and industrial applications.

## Figures and Tables

**Table 1:** Comprehensive comparison among the five discussed computational techniques.

Technique	Scale	Method	Advantages	Limitations
DFT	Hundreds to a few thousand atoms over short time scales	Solves Schrödinger’s equation (quantum mechanics)	Provides highly accurate predictions of electronic structure and bonding properties	Computationally intensive and limited to relatively small systems
MD	Thousands to millions of atoms over nanosecond–microsecond time scales	Solves Newton’s equations of motion (classical mechanics)	Captures atomistic dynamics with lower cost than DFT; suitable for studying thermal, mechanical, and transport properties	Accuracy depends on interatomic potentials; limited for systems with complex chemistry or long-time phenomena
CGMD	Millions to billions of coarse-grained beads over microsecond–millisecond time scales	Groups atoms into coarse-grained beads to reduce degrees of freedom	Extends accessible spatial and temporal scales while retaining essential physics; ideal for mesoscale deformation and self-assembly studies	Loses atomic-level detail; coarse-graining potentials require extensive calibration and validation
FEM	Continuum to macroscopic scale	Discretizes the domain into finite elements to solve governing continuum equations	Efficient for simulating large-scale mechanical behavior and structural deformation	Inapplicable at atomic scales; limited accuracy for highly nonlinear or heterogeneous systems
ML	Not limited by scale; determined by the scope of training data	Data-driven approach that learns relationships among input features	Extremely fast once trained; integrates and interprets data from DFT, MD, CGMD, and FEM; capable of exploring uncharted parameter spaces	Strongly dependent on training data quality; limited interpretability; model hyperparameter tuning can be challenging

## Data Availability

No new data were created or analyzed in this study.

## Abbreviations

Machine learning (ML), Density Functional Theory (DFT), Molecular Dynamics (MD), Artificial Intelligence (AI), Coarse-Grained (CG), All-Atom (AA), Finite Element Modeling (FEM), Artificial Neural Network (ANN), Gaussian Process (GP), and Support Vector Machine (SVM), steered molecular dynamics (SMD), Monte Carlo (IMC), iterative Boltzmann inversion (IBI), and force matching (FM), K-nearest neighbors (KNN), cellulose nano fiber micro-fibrils (CNF-MFs), discrete element method (DEM), tetrabutylphosphonium chloride (TBPCl), calcium-silicate-hydrate (C-S-H), carbon nanotube (CNT), polyethylene (PE), cellulose nano crystal (CNC), polylactic acid (PLA), supra-CG (sCG), atomic force microscopy (AFM), Doum Palm Leaf Stalk Fiber (DPLSF), Shapley Additive exPlanations (SHAP), cellulose nanofiber (CNF), specific surface area (SSA), convolutional neural network (CNN), glass transition temperature (Tg), carbohydrate-binding domain (CBD), mean square displacement (MSD), micro-computed tomography (μCT), Near-Infrared (NI), cross-laminated timber (CLT), Finite Volume Method (FVM), Representative Elementary Volume (REV), higher heating value (HHV), thermogravimetric analysis (TGA), Sequential Regression Method (SRM), differential thermogravimetric (DTG), random forest (RF), electrostatic potential (ESP), overall average value (OAV), positive average value (PAV), density of states (DOS), van der Waals (vdW), Quantum mechanical (QM), Projected density of states (PDOS), bacterial cellulose (BC), time-dependent DFT (TDDFT), ionic liquids (ILs), 5-hydroxymethylfurfural (5-HMF), natural bond orbitals (NBO), hydrogen bond (HB), lifecycle assessment (LCA).
